# Unravelling the mechanism of cobalt-catalysed remote C–H nitration of 8-aminoquinolinamides and expansion of substrate scope towards 1-naphthylpicolinamide†
†Electronic supplementary information (ESI) available: Experimental; experimental protocols, characterization data, and NMR spectra of all new compounds. DFT studies; details of methods used and coordinates for all complexes. See DOI: 10.1039/c9sc05076k


**DOI:** 10.1039/c9sc05076k

**Published:** 2019-11-18

**Authors:** Melody Chu, Oriol Planas, Anna Company, Xavi Ribas, Alex Hamilton, Christopher J. Whiteoak

**Affiliations:** a Department of Biosciences and Chemistry , Sheffield Hallam University , Sheffield , S1 1WB , UK . Email: c.whiteoak@shu.ac.uk ; Email: a.hamilton@shu.ac.uk; b Departament de Química , Grup de Química Bioinspirada, Supramolecular i Catàlisi (QBIS-CAT) , Institut de Química Computacional i Catàlisi (IQCC) , Universitat de Girona , Campus de Montilivi , 17071 Girona , Catalonia , Spain

## Abstract

Previously, an unexpected Co-catalysed remote C–H nitration of 8-aminoquinolinamide compounds was developed. This report provided a novel reactivity for Co which was assumed to proceed through the mechanistic pathway already known for analogous Cu-catalysed remote couplings of the same substrates. In order to shed light into this intriguing, and previously unobserved reactivity for Co, a thorough computational study has now been performed, which has allowed for a full understanding of the operative mechanism. This study demonstrates that the Co-catalysed remote coupling does not occur through the previously proposed Single Electron Transfer (SET) mechanism, but actually operates through a high-spin induced remote radical coupling mechanism, through a key intermediate with significant proportion of spin density at the 5- and 7-positions of the aminoquinoline ring. Additionally, new experimental data provides expansion of the synthetic utility of the original nitration procedure towards 1-naphthylpicolinamide which unexpectedly appears to operate *via* a subtly different mechanism despite having a similar chelate environment.

## Abstract 2

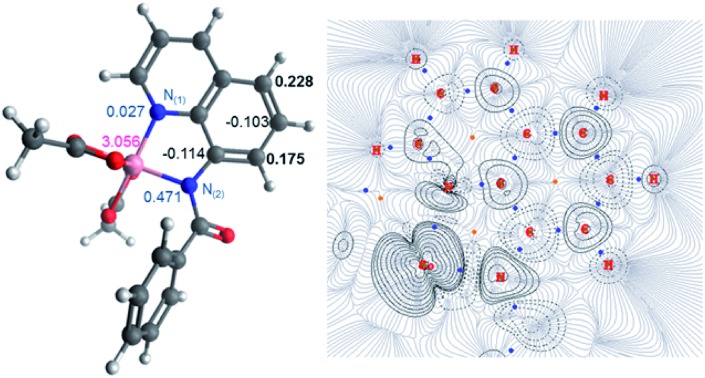
Computational studies of the Co-catalyzed remote nitration of 8-aminoquinolinamides have found the mechanism to operate through an unexpected high-spin induced remote radical-coupling.

## Introduction

The controlled and selective functionalisation of C–H bonds is currently a topic which is attracting a significant amount of interest from the chemical community.^1^ When successfully applied, C–H functionalisation protocols are generally used to either reduce the length of complex multi-step syntheses or to provide rapid access to new derivatives of biologically active molecules as a late stage functionalisation tool.^2^ The challenge in the latter application is to regiochemically functionalise a complex compound in a predictable and controlled fashion, whereby there are often a number of other reactive functional groups present. Currently, a majority of the developed C–H functionalisation protocols utilise a chelation directing group strategy for control of product selectivity^3^ and well established/understood Concerted Metalation Deprotonation (CMD) or Oxidative Addition (OA) steps.^4^ These key mechanistic approaches are very common when employing second and third-row transition metals in C–H functionalisation. In contrast, the chemistries of first-row transition metals are more diverse than second- and third-row analogues, which provides an exciting potential opportunity to discover and develop novel protocols outside of those using traditional CMD and OA steps (*e.g.* radical coupling approaches).^5^ In the case of late stage C–H bond functionalisation, these unique reactivities could be applied to provide access to previously inaccessible analogues of biologically active compounds through new innovative C–C and C–X bond forming protocols.

Several first-row transition metals have been shown to provide reactivities amenable for application in C–H functionalisation protocols. However, the already known rich mechanistic diversity of Co in the field of C–H functionalisation makes this metal stand out as an exciting candidate for the basis of novel protocols.^6^,^7^

Functionalisation of quinolines beyond traditional C2 modification has recently become a topic of increasing interest^8^ as quinoline is an important heterocycle found in many biologically active compounds. For example, the 8-aminoquinoline scaffold is the basis of a number of anti-malarial drugs^9^ and facile modification as a late stage functionalisation tool may provide access to new potent drugs which could have significant impact on one of the world's major causes of death.^10^

**Scheme 1:**
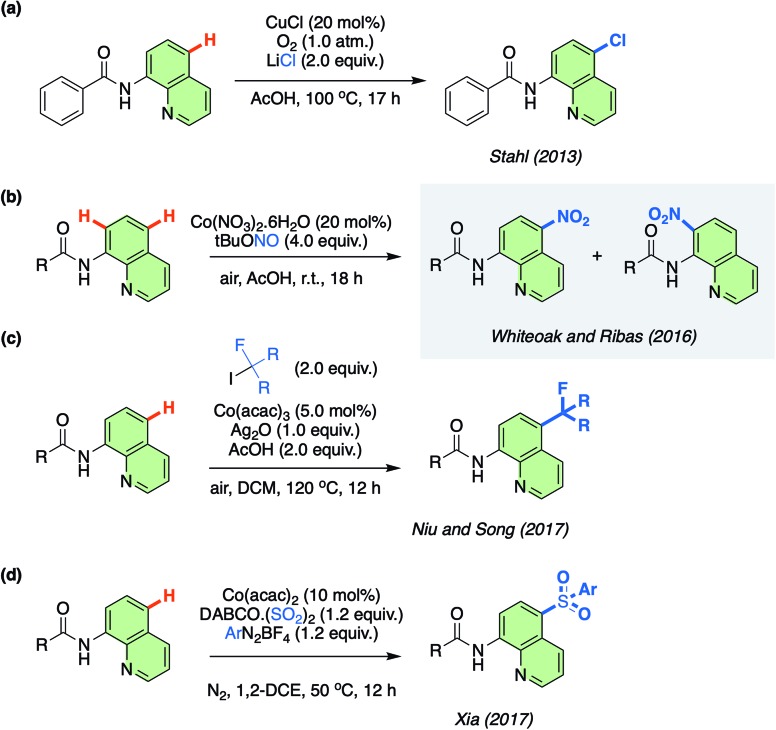
(a) Cu-catalysed remote chlorination reported by Stahl. (b) First Co-catalysed remote nitration reported by Whiteoak and Ribas. (c) Co-catalysed remote perfluoroalkylation reported by Niu and Song. (d) Co-catalysed remote sulfonylation reported by Xia.

One novel approach to the functionalisation of 8-aminoquinolinamides has been the remote (non-proximate) C–H bond chlorination catalysed by Cu at the C7 position, which was originally reported by Stahl/Ertem and co-workers in 2013 (Scheme 1a).^11^ Non-proximate C–H bond functionalisation, therefore moving beyond directing group based protocols, is a major challenge in synthetic chemistry with only a limited, but increasing, number of approaches currently available.^12^ This initial work by Stahl/Ertem and co-workers demonstrated the first example of a metal-catalysed remote C–H functionalisation of 8-aminoquinolinamides and Density Functional Theory (DFT) calculations were employed to elucidate the mechanism, which was found to be based on a key Single Electron Transfer (SET) step. This seminal work has been the inspiration for the development of an ever-expanding number of practical Cu-catalysed remote C–H functionalisation protocols of the same substrate.^13^ Meanwhile, other metals have also been employed as catalysts for remote C–H functionalisation of these 8-aminoquinolinamides, although to a significantly reduced extent.^14^

Resulting from the rich aforementioned chemistry of Co, in 2016 some of us reported on the unexpected remote nitration of 8-aminoquinolinamide scaffold using *tert*-butyl nitrite (TBN) as nitro source (Scheme 1b).^15^,^16^ This was the first time that Co had been observed to display this type of reactivity. Since this report, others have also been attracted to the potential of this novel potential for Co in the field of C–H functionalisation (Scheme 1c and d).^17^ The lack of understanding of this new Co-catalysed approach has now inspired us to examine the mechanism in more detail utilising the open shell DLPNO-CCSD(T) (Domain-based Local Pair-Natural Orbital Coupled Cluster approximations) method, in order that the unique reactivity of Co can be fully understood and as a result be further rationally exploited by others.

Herein, we describe our findings and also demonstrate that the protocol can also be successfully transferred to the remote nitration of 1-naphthylpicolinamide analogues which replace the aminoquinolinamide chelate environment with a picolinamide, thus further expanding the applicability of the previously discovered protocol.

## Results and discussion

The initial approach in this study was to identify the nature of the starting “Co(ii) source” in the mechanism. Given the use of AcOH as solvent, the experimental conditions would allow for extensive counter-ion ligand exchange, leading to a number of potential Co(ii) species. Relative free energies for ligand exchange (Table 1) identified Co(OAc)_2_ as the most thermodynamically stable Co species. This compound was therefore used as the basis for the active catalyst species for the remainder of the reaction mechanism. It should be noted at this point that the observed benefit of using Co(NO_3_)_2_ over Co(OAc)_2_ in the original protocol^15^ is likely a result of the background nitration of the substrate through traditional nitration utilising *in situ* formed HNO_3_ in an acidic environment.

**Table 1:** Relative free energy, Δ*G*_298_ kcal mol^–1^, for Co(ii) species

Co(ii) species	Relative free energy, Δ*G*_298_ (kcal mol^–1^)
Co(NO_3_)_2_	0.00
Co(NO_3_) (OAc)	–20.21
Co(OAc)_2_	–48.34

After this initial study on catalyst species we embarked on full elucidation of the reaction mechanism. As a result of the moderate to large size of the catalytic system being studied, DFT calculations would traditionally be the method of choice. However, due to the potential for different spin states to be involved in the mechanism, which pose a significant challenge for DFT based methods, the recently implemented open shell DLPNO-CCSD(T) method was utilised.^18^,^19^ This near linear scaling *ab initio* electronic structure method has the potential for coupled cluster accuracy for large, synthetically relevant catalysts. Recent work by Chen and co-worker on the same catalyst, but focusing on a different C–H activation protocol, highlighted the importance of these methods due to the complicated multi-state reactivity which has been previously reported.^20^ In this report Multi-State Reactivity (MSR) was found to be an intriguing feature of the chemistry of high-spin Co(iii), where this report revealed the highly complex mechanisms operative in Co-catalyzed C–H activation processes.

**Fig. 1:**
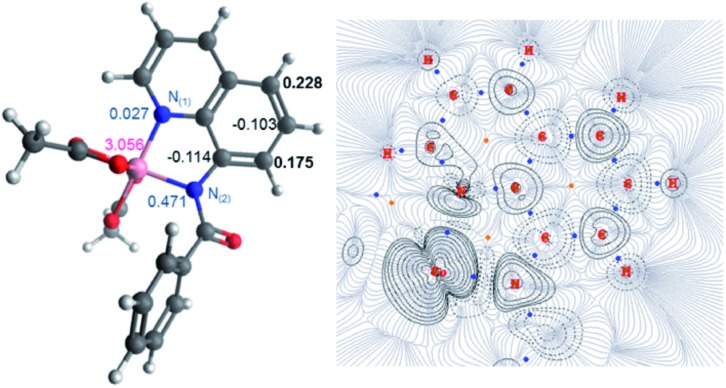
(Left) Molecular structure with calculated spin densities for **^5^Int 1·L^1^** obtained using DLPNO-CCSD/def2-tzvp highlighting the spin accumulated at the 5- and 7-positions on the 8-aminoquinolinamide ligand. (Right) QTAIM wavefunction analysis of spin density gradients and bond critical points, focusing on the 8-aminoquinoline moiety.

The first step of the reaction is hydrogen abstraction by the *in situ* formed ^*t*^BuO˙ radical leading to the radical of the 8-aminoquinolinamide ligand, **L^1^**. Coupling **L^1^** to the [Co(AcO)_2_] pre-catalyst forms the initial catalytic species **Int1·L^1^**. This species can exist in three possible spin states; low spin singlet (**^1^Int 1**, *S* = 0), medium spin triplet (**^3^Int 1**, *S* = 1) and high spin quintet (**^5^Int 1**, *S* = 2). With the DLPNO-CCSD(T) method the high spin quintet state (**^5^Int 1·L^1^**) proved to be the ground-state species, which is in full agreement with previous work from Chen and co-worker.^20^ Examination of the electronic structure of the three potential spin state complexes leads to the conclusion that both **^1^Int 1·L^1^** and **^3^Int 1·L^1^** are Co(iii) complexes whereas the **^5^Int 1·L^1^** is actually a high-spin quartet Co(ii) species with the radical still residing on the quinoline moiety of the ligand. Cumulative spin densities on the aminoquinoline ligand, calculated using DLPNO-CCSD/def2-tzvp,^21^ for **^1^Int 1·L^1^**, **^3^Int 1·L^1^** and **^5^Int 1·L^1^** are 0, 0.18 and 0.72, respectively. Fig. 1 highlights the increased electron spin density residing at 5- and 7-positions, showing the potential sites for radical addition to **^5^Int 1·L^1^**. A similar spin population distribution was previously observed by Chen and co-worker leading to the description as a quartet Co(ii) ferromagnetically coupled with a ligand radical.^20^

Topological analysis using Quantum Theory of Atom in Molecules (QTAIM)^22^ and different bond order descriptors (Table 2) for the three spin state structures highlight a significant decrease in bonding/interaction between the Co centre and the 8-aminoquinolinamide ligand on progression from singlet to quintet state. This is in further agreement with the assignment of **^1^Int 1·L^1^** and **^3^Int 1·L^1^** being Co(iii) whereas **^5^Int 1·L^1^** being high spin Co(ii) with an associated radical ligand, based on the spin density analysis.

**Table 2:** Bonding and QTAIM parameters for different spin-states of **Int1·L^1^**^*a*^

Bonding parameter	**^1^Int1·L^1^**		**^3^Int1·L^1^**		**^5^Int1·L^1^**	
	Co–N_(1)_	Co–N_(2)_	Co–N_(1)_	Co–N_(2)_	Co–N_(1)_	Co–N_(2)_
*r*	1.948	1.894	1.878	1.925	1.985	2.073
*ρ*	0.104	0.113	0.125	0.108	0.087	0.072
∇^2^*ρ*	0.408	0.563	0.441	0.474	0.477	0.356
*H*(*r*)	–0.030	–0.031	–0.045	–0.032	–0.020	–0.014
FBO	1.053	1.010	1.183	0.982	0.901	0.784
MBO	0.736	0.651	0.777	0.626	0.434	0.399
LBO	0.369	0.515	0.450	0.454	0.343	0.268

^*a*^Bonding parameters: Co–N_(1)amide_ Co–N_(2)pyrrole_: distance *r* in Å, QTAIM parameters in a.u.; *ρ* electron density, ∇^2^*ρ* laplacian of electron density, *H*(*r*) local energy density. FBO = Fuzzy Bond Order,^24^ MBO = Mayer Bond Order,^25^ LBO = Laplacian Bond Order.^26^

**Fig. 2:**
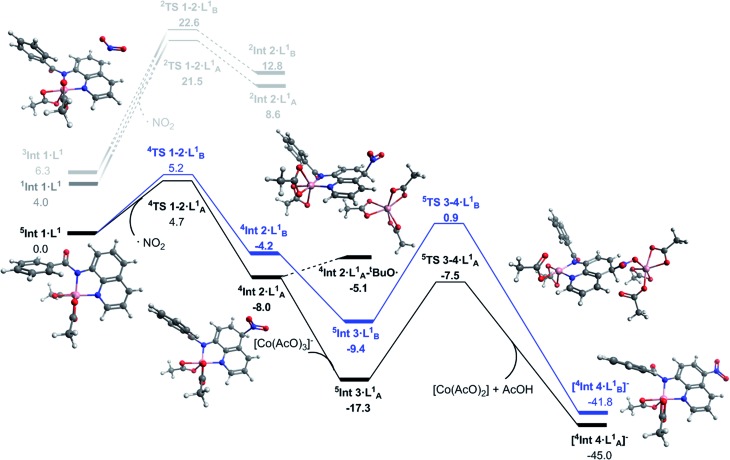
Solvated free energy surface (Δ*G*_298_) for the nitration of 8-aminoquinolinamide calculated with DLPNO-CCSD(T)/def2-TZVP. Black (A) and blue (B) lines represent high spin nitration at the 5 and 7 positions respectively. Dark grey represents first excited state optimised geometry of **Int 1·L^1^**. Light grey represents the corresponding low spin pathway. Calculated geometries shown are for the observed 5-position nitration *via* the high spin mechanism.

The transition states for the remote NO_2_ radical coupling at the 5- and 7-positions starting from **^5^Int 1·L^1^** lie at 4.7 and 5.2 kcal mol^–1^ respectively (Fig. 2). This low barrier height is in good agreement with the protocol being operative under ambient conditions. Additionally, the nitration step is also the regioselective transition state with a ΔΔ*G*‡298 K of 0.5 kcal mol^–1^ in favour of the observed 5-substituted product. This difference results in a calculated regioselectivity of 60%, which is in reasonably good agreement with the experimentally observed 3 : 1 product ratio. Calculation of the low spin nitration reaction, *via***^2^TS 1–2·L_A/B_^1^**, showed it to be approximately 17 kcal mol^–1^ less favourable than the proposed high spin mechanism shown in Fig. 2. This substantial stabilisation of the high-spin mechanism further strengthens the proposed remote radical coupling mechanism *via* the quintet state. There has been discussion in the literature regarding the potential over-stabilisation of high spin complexes with DLPNO-CCSD(T) methods,^23^ however the degree of over-stabilisation is in the order of 5 kcal mol^–1^ at most and therefore as a result we are confident this reaction proceeds *via* the proposed high spin pathway.

To ensure the previously proposed intramolecular Singlet Electron Transfers (SET) mechanism was not involved in the reaction, LR-TD-DFT (Linear Response Time Dependent-Density Functional Theory) calculations were performed on **^1^Int 1·L^1^**. The first excited state transition, corresponding to the excitation of an electron from the 8-aminoquinolinamide ligand to the Co(iii) centre, is found to be in the range of 37–43 kcal mol^–1^, depending on the choice of DFT functional and amount of HF exchange included (see ESI for full details†). The transition energy is similar to that reported by Stahl/Ertem and co-workers for the related Cu(ii) complex.^11^ Optimisation of the corresponding excited state structure, **^1^Int 1_ES_·L^1^**, with the hybrid functional PBE0 provides a value of 24.4 kcal mol^–1^ above **^1^Int 1·L^1^**. Although not directly comparable due to the differing methodologies used, both the transition energy and the optimised excited state complex is significantly higher in energy than the transition state on the proposed high-spin mechanism starting from the quintet ground state, **^5^Int 1·L^1^**. As a result of the requirement for a coupling transition state after **^1^Int 1_ES_·L^1^**, this precludes this mechanism on the grounds of it being energetically unfeasible.

Continuing with the high-spin mechanism, the nitration of **^5^Int 1·L^1^** results in the formation of the intermediate complex, **^4^Int 2·L^1^**. This intermediate is 8.0 kcal mol^–1^ lower in energy than the preceding starting complex. The lower energy pathway leading to nitration at the 5-position forms **^4^Int 2·L_A_^1^** which is 3.8 kcal mol^–1^ more stable than the corresponding 7-substituted product (**^4^Int 2·L_B_^1^**), and 16.6 kcal mol^–1^ more stable than the equivalent low spin doublet mechanism (**^2^Int 2·L_A_^1^**). A number of different pathways for the deprotonation of **^4^Int 2·L^1^** at the 5- and 7-positions were explored. Addition of [Co(AcO)_3_]^–^ to **^4^Int 2·L^1^** forms **^5^Int 3·L^1^**, this step is exergonic by 9.7 kcal mol^–1^. Cobalt assisted deprotonation by acetate anion has a barrier of 9.8 kcal mol^–1^, leading to the final intermediate **[^4^Int 4·L^1^]^–^**, which undergoes proto-demetallation to form the observed product and regenerates the catalyst, completing the catalytic cycle. Attempts to optimise the transition state for deprotonation by an isolated acetate anion led directly to **[^4^Int 4·L^1^]^–^**, suggesting this pathway could also be occurring, but no energetic barrier is reported. Due to the reaction conditions allowing for the formation of ^*t*^BuO˙ (eqn (1)), a hydrogen atom abstraction pathway was also explored. Formation of the adduct complex **^4^Int 2·L_A_^1^**–^*t*^BuO˙ is endergonic by 2.9 kcal mol^–1^ and therefore 12.2 kcal mol^–1^ less favourable than **^5^In t3·L_A_^1^** and 2.4 kcal mol^–1^ less favourable than **^5^TS 3–4·L^1^**. Based on these results we propose cobalt assisted acetate deprotonation is the active pathway (Fig. 2). Nitration at the 7-position (route B) is consistently between 3 and 8 kcal mol^–1^ less favourable throughout the entire mechanism, and in good agreement with the experimental observations for regioselectivity.

1 ^*t*^BuONO → ^*t*^BuO˙ + NO

2
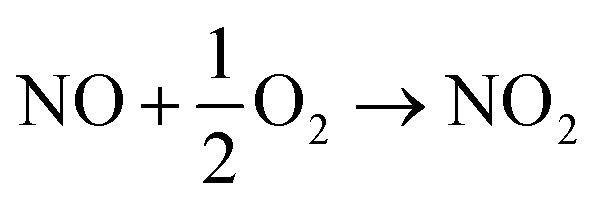



**Scheme 2:**
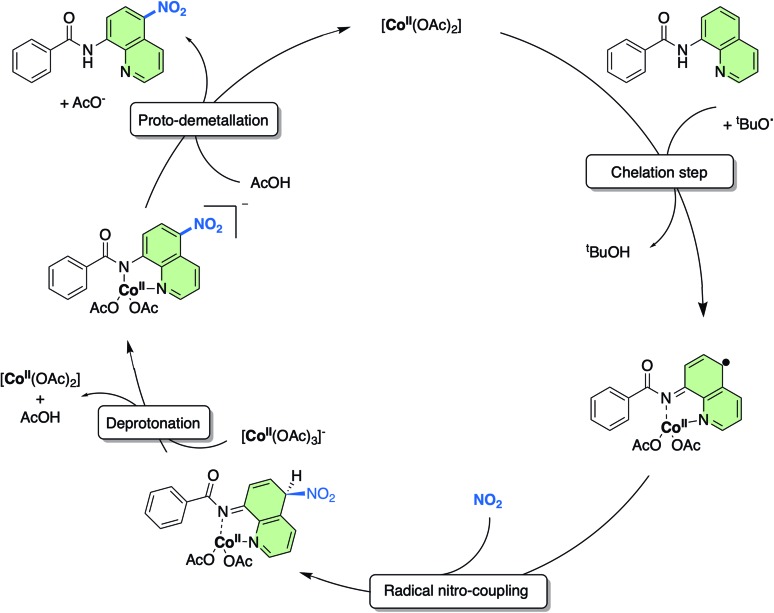
Proposed mechanism for the remote nitration of 8-aminoquinolinamide substrates catalysed by Co(OAc)_2_ at the 5-position based on the computational studies in from this work. Note: the NO_2_ is formed *in situ* from the decomposition of TBN (see eqn (1) and (2)).

As a result of this new in-depth study, in adjustment to our previously proposed SET mechanism for the Co-catalysed remote nitration of 8-aminoquinolamide substrates, we now propose the mechanism shown in Scheme 2. In the first step, the Co(ii) coordination to substrate is promoted by H˙ abstraction from the amide by the ^*t*^BuO˙ radical generated from *in situ* decomposition of the TBN. This results in a high spin Co(ii) species with partially delocalised radical character on the 5- and 7-positions of the 8-aminoquinolinamide moiety which then reacts with the nitrogen dioxide (NO_2_) formed from the decomposition of TBN (eqn (1) and (2)).^27^ Deprotonation of the intermediate coupling product with the *in situ* formed [Co(OAc)_3_]^–^ furnishes the chelate species of the final product, which can then undergo proto-demetallation by acetic acid to reform the initial Co(OAc)_2_ catalyst, releasing the final nitrated 8-aminoquinolinamide product.

**Scheme 3:**
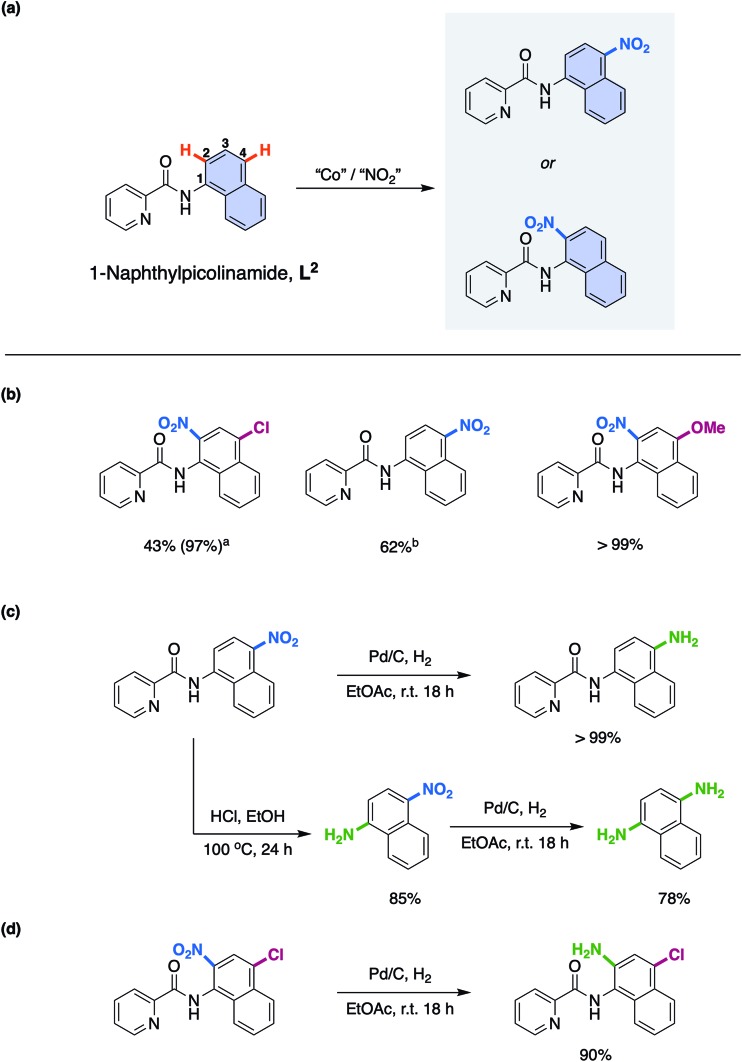
(a) 1-Napththylpicolinamide substrates used in the experimental work; nitration at either the 2- or 4-position for the 1-napththylpicolinamide. (b) Products isolated from the Co-catalysed remote nitration of 1-napththylpicolinamide substrates; reaction conditions: 0.50 mmol 1-napththylpicolinamide substrate, 20 mol% Co(NO_3_)_2_·6H_2_O, 4.0 equivalents TBN, 3.5 mL AcOH, r.t., 18 h. ^*a*^6.0 equivalents of TBN. ^*b*^Only the 4-substituted product could be successfully isolated, and the yield shown is of this regioisomer. (c) Examples of product upgrading of the nitrated compound obtained from 1-naphthylpicolinamide. (d) Examples of product upgrading of the nitrated compound obtained from 4-chloro-substituted 1-naphthylpicolinamide substrate.

With this understanding in hand, we turned our attention to expanding the scope of this novel reaction mechanism. Rather than focus on addition of different coupling partners at the 5- and 7-positions of the 8-aminoquinolinamide substrate, something which has already been exemplified by both Xia and co-workers17*a* and Niu and Song and co-workers,17*b* we choose to focus on selective nitration of substrates that have proven more challenging by more traditional synthetic methods. In an effort to direct experiments a new substrate was rationally proposed which would facilitate selective 1-aminonaphthyl nitration while maintaining the key bi-dentate nitrogen chelation. 1-Naphthylpicolinamide, **L^2^**, (Scheme 3a) was chosen to ensure formation of the proposed required Co chelate intermediate. Indeed, Cu examples of remote functionalisation of these substrates are known and have previously been reported.^28^

As with the 8-aminoquinolinamide substrate, it was possible to convert a number of differently substituted 1-naphthylpicolinamide substrates to the corresponding nitrated products as shown in Scheme 3b. The electron-withdrawing 4-chloro-substituted 1-naphthylpicolinamide, provided clean nitration at the 2-position. It should be noted that in order to get an almost quantitative yield (97%), it was necessary to increase the TBN loading from 4.0 equivalents to 6.0 equivalents.^15^ The lower reactivity of electron-withdrawing substituted substrates was previously observed with the 8-aminoquinolinamide substrates.^15^ The parent 1-naphthylpicolinamide substrate with no substituents could also be nitrated, although despite our best efforts it was not possible to successfully isolate the 2-nitration product. As a result of this, the reported yield therefore corresponds to the 4-nitration product only, which was successfully obtained as an analytically pure sample. Finally, the electron-donating methoxy-substituted substrate could also be cleanly converted to the corresponding 2-nitrated product quantitatively.

In order to exemplify the potential for these compounds for further utility, the 1-naphthylpicolinamide with nitration at the 4-position was initially reduced to the corresponding amine using Pd/C and H_2_ (Scheme 3c). Additionally, the picolinamide bond could be hydrolysed under acid conditions to furnish 1-amino-4-nitronaphthalene in good yield (85%) and thereafter, the 1,4-diaminonaphthalene was prepared in good yield through reduction of this nitro-product using Pd/C and hydrogen (78%; Scheme 3c). Finally, a highly substituted product containing synthetically useful chloro and amine functionalities again obtained through a facile Pd/C and H_2_ reduction of the nitration product of the 4-chloro-substituted 1-naphthylpicolinamide could be easily prepared (Scheme 3d).

**Fig. 3:**
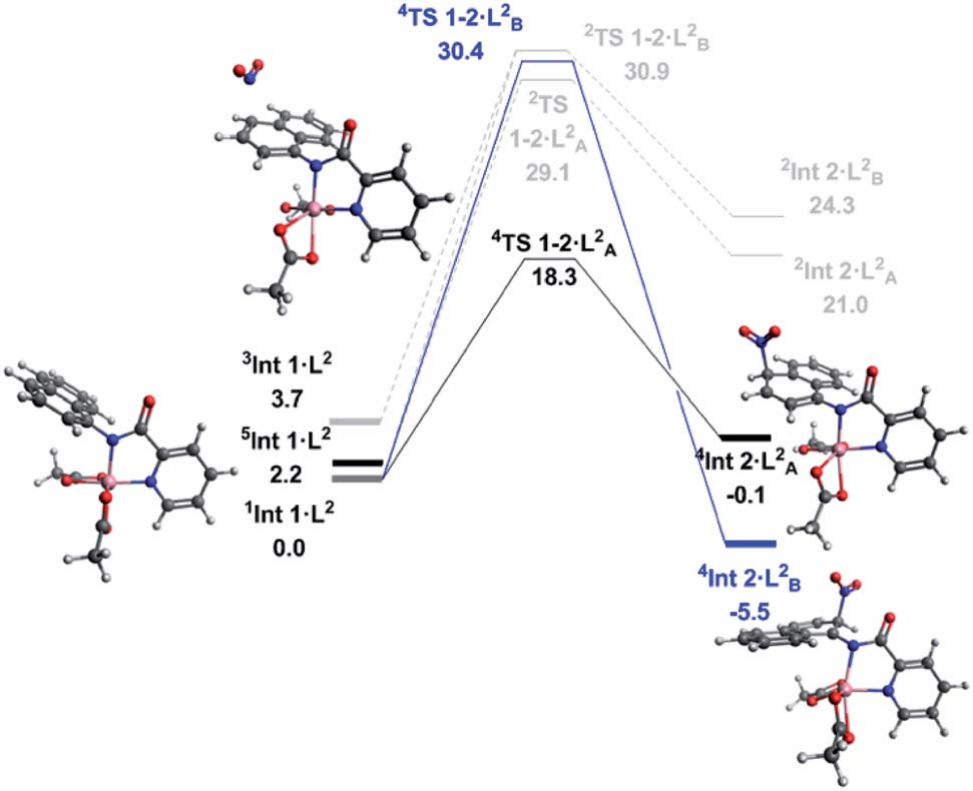
Solvated free energy surface (Δ*G*_298_) for the nitration of 1-naphthylpicolinamide calculated with DLPNO-CCSD(T)/def2-TZVP. Black (A) and blue (B) lines represent high spin nitration at the 4 and 2 positions respectively. Dark grey represents first excited state optimised geometry of Intermediate 1. Light grey represents the corresponding low spin pathways. Calculated geometries shown are for the observed 4-position nitration *via* the high spin mechanism, as well as the intermediate for the 2-position nitration.

With successful transfer of the original nitration reaction to the 1-naphthylpicolinamide substrates we wondered if it followed the same reaction mechanism. Initially it was assumed that the 1-naphthylpicolinamide substrate would follow a similar reaction path to the 8-aminoquinolinamide example. In contrast to the 8-aminoquinalineamide substrate, the DLPNO-CCSD(T) calculations suggest the ground state of the 1-naphthylpicolinamide complex is the singlet (*S* = 0) with the quintet (*S* = 2) and triplet (*S* = 1) states being 2.2 and 3.7 kcal mol^–1^ higher in energy respectively (Fig. 3). TDDFT calculations on **^1^Int 1·L^2^** gives the first excited state transition, corresponding to the SET mechanism, of 37.7 kcal mol^–1^. An equivalent to the barrier to this was seen for **^1^Int 1·L^1^**.

**Fig. 4:**
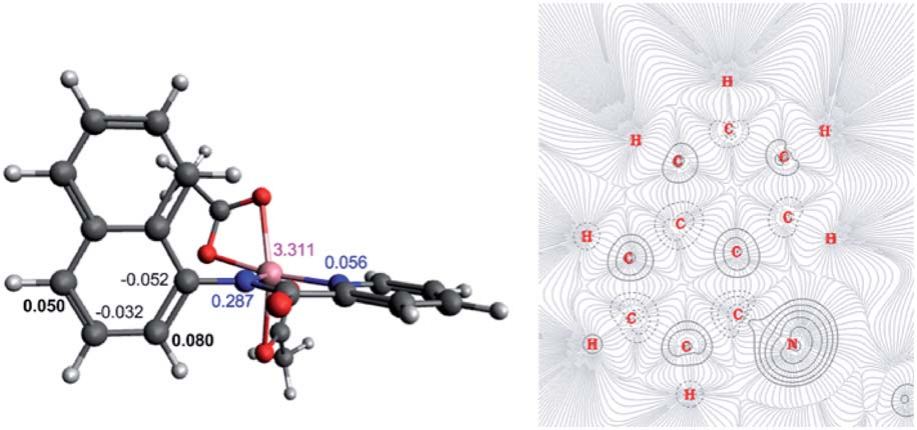
(Left) Molecular structure with calculated spin densities for **^5^Int 1·L^2^** obtained using DLPNO-CCSD/def2-tzvp highlighting the spin accumulated at the 2- and 4-positions on the 1-aminonaphthyl moiety. (Right) QTAIM wavefunction analysis of spin density gradients, focusing on the 1-aminonaphthyl moiety.

Thereafter spin density analysis of **^5^Int 1·L^2^** showed a significant proportion of density at the observed reactive 2- and 4-positions of the 1-aminonaphthyl moiety (Fig. 4), again highlighting the potential for remote radical coupling. Nitration at the 2- and 4-position of the 1-naphthylpicolinamide substrate can then proceed *via* either a low spin (*S* = 1/2) or high spin (*S* = 3/2) pathway. As with the 8-aminoquiolinamide mechanism the high spin pathway, through **^4^TS 1-2·L^2^**, is significantly more favourable. This suggests a Multi-State Reactivity (MSR) mechanism with crossing between potential energy surfaces,^29^ from low to high spin, as the nitration step proceeds in order to facilitate the remote radical coupling. The high spin products of the nitration step, **^4^Int 2·L_A/B_^2^**, are approximately 25 kcal mol^–1^ more energetically stable compared to the equivalent low spin intermediate, **^2^Int 2·L_A/B_^2^**. Interestingly the observed substitution at the 4-position is kinetically favoured, while the 2-position is the thermodynamic intermediate. This can be accounted for by the additional long range hydrogen bonding (2.45 Å) between the hydrogen at the 2-position of the nitrated naphthyl moiety and the Co bound acetate. The calculated higher energy barriers for 1-naphthylpicolinamide compared to 8-aminoquinolinamide agrees with the experimental requirement for more forcing reaction conditions (increase in number of equivalents of TBN in the case of 4-chloro-substituted 1-naphthylpicolinamide). Increased spin density on the Co and decreased spin density on the 1-aminonaphthyl moiety further support this decrease in reactivity (Fig. 4).

## Conclusions

In summary, we have studied the mechanism of the remote nitration of 8-aminoquinolinamides catalysed by Co using the robust DLPNO-CCSD(T) methodology and found there to be a significant difference in mechanism to the well-studied analogous Cu-catalysed remote functionalisation protocols of 8-aminoquinolinamides. The results reported within this work identify an unusual mechanism which does not pass through the initially proposed SET process, but instead, the reaction takes place *via* a High-Spin Remote Induced Radical Coupling, providing an alternative route for the NO_2_ to couple compared with a traditional SET process. The high spin reactivity has been successfully transferred to 1-naphthylpicolinamide substrates, further enhancing the applicability of the original protocol, where the computational study has demonstrated that a subtly different mechanism is operative (Multi-State Reactivity). Overall, the distinct mechanistic pathways shown in this work provide a complimentary tool for the remote C–H functionalisation of both 8-aminoquinolinamides and 1-naphthylpicolinamides. This new mechanistic understanding should now inspire others to develop new synthetic protocols for remote functionalisation using Co-catalysis.

## Conflicts of interest

There are no conflicts to declare.

## Supplementary Material

Supplementary information
