# Supplementary material for: Unravelling the mechanism of cobalt-catalysed remote C–H nitration of 8-aminoquinolinamides and expansion of substrate scope towards 1-naphthylpicolinamide
Source: Chem Sci. 2019 Nov 18;11(2):534–42. doi: 10.1039/c9sc05076k (PMC7067239; doi:10.1039/c9sc05076k)
Supplement: Supplementary file 1 [file SC-011-C9SC05076K-s001.pdf]

Electronic Supplementary Material (ESI) for Chemical Science.
This journal is © The Royal Society of Chemistry 2019

Supporting information for:

Unravelling the Mechanism of Cobalt-Catalysed Remote C-H Nitration of 8-Aminoquinolinamides and Expansion of Substrate Scope Towards 1-naphthylpicolinamide

Melody Chu,[a] Oriol Planas,[b] Anna Company,[b]
Xavi Ribas,[b] Alex Hamilton[a]* and Christopher J. Whiteoak[a]*

[a] Department of Biosciences and Chemistry, Sheffield Hallam University, Sheffield, S1 1WB, United Kingdom.

[b] Grup de Química Bioinspirada, Supramolecular i Catàlisi (QBIS-CAT), Institut de Química Computacional i Catàlisi (IQCC) and Departament de Química, Universitat de Girona, Campus de Montilivi, 17071 Girona, Catalonia, Spain

Contents:

Page S2.............................. [1] GENERAL EXPERIMENTAL CONSIDERATIONS

Page S2.............................. [2] GENERAL PROCEDURE FOR NITRATION REACTIONS

Page S2.............................. [3] SYNTHESIS AND CHARACTERIZATION DATA FOR PRODUCTS

Page S6.............................. [4] ORIGINAL SPECTRA FOR NEW PRODUCTS

Page S13............................ [5] COMPUTATIONAL DETAILS

Page S16............................ [6] COORDINATES FROM COMPUTATIONAL STUDY

Page S30............................ [7] REFERENCES

[1] General Experimental Considerations:

All reagents and solvents were purchased from Sigma Aldrich, Fisher Scientific and Acros Organics and used without further purification. ^{1}H (400 MHz), $^{13}C\ \{^{1}H\}$ (100 MHz), $^{19}F\ \{^{1}H\}$ (376 MHz) and COSY NMR were recorded on a Bruker AV-400 spectrometer and referenced to the residual deuterated solvent signals. High Resolution Mass Spectra (HRMS) was recorded on a Xevo G2-Xs QTof Mass Spectrometer. 1-Napththylpicolinamide substrates used in this study were prepared by literature methodologies.[1-3]

[2] General Procedure for Nitration Reactions:

The procedure for the conversion of the 1-napththylpicolinamide substrates to the corresponding nitrated products is the same as that previously reported for the 8-aminoquinolinamide substrates;[4]

A 10 mL vial was charged with 0.5 mmol of substrate, $Co(NO_3)_2.6H_2O$ (29.1 mg, 20 mol%, 0.1 mmol), tert-butyl nitrite (267 µL, 90%, 4.0 equiv., 2.0 mmol) and 3.5 mL of acetic acid. The vial was then sealed, and the reaction stirred at room temperature for 18 hours. After this period the reaction mixture was diluted with ethyl acetate (30 mL) and extracted using brine (20 mL). The aqueous layer was further extracted with ethyl acetate (2 x 30 mL), the organic layers combined, dried over magnesium sulfate and the solvent removed under reduced pressure. The crude reaction mixture was purified by column chromatography, using ethyl acetate/dichloromethane as eluent, providing analytically pure nitrated products.

[3] Synthesis and Characterization Data for Products:

N-(4-nitro-1-naphthalenyl)-2-pyridinecarboxamide:

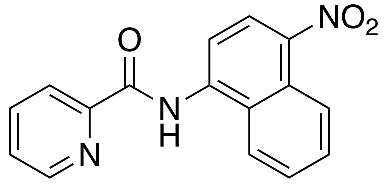

This compound was made according to the general procedure for nitration reaction described above starting from 130 mg, (0.5 mmol) of substrate to yield a pale yellow crystalline solid (91 mg, 62 %). ^{1}H NMR ($CDCl_3$, 400 MHz, 298 K); δ = 11.25, (br s, 1H), 8.84-8.78 (m, 2H), 8.75 (d, 1H, $^{3}J_{HH}$ = 8.3 Hz), 8.46-8.38 (m, 2H), 8.26 (d, 1H, $^{3}J_{HH}$ = 8.3 Hz), 8.04 (ddd, 1H, $^{3}J_{HH}$ = 7.7, $^{3}J_{HH}$ = 7.7, $^{4}J_{HH}$ = 1.5 Hz), 7.85-7.77 (m, 2H), 7.66-7.60 (m, 1H). $^{13}C\{^{1}H\}$ NMR ($CDCl_3$, 100 MHz, 298 K); δ = 162.34, 149.28, 148.33, 142.31, 138.40, 138.12, 129.46, 127.57, 127.22, 126.36, 125.86, 125.67, 124.42, 122.81, 120.57, 114.83. HR-MS (ESI, m/z); calcd. for $C_{16}H_{11}N_3O_3$+H = 294.0879; obtained = 294.0873 $[M+H]^+$. R_f = 0.80 (ethyl acetate/dichloromethane = 1/3).

N-(2-nitro-4-chloro-1-naphthalenyl)-2-pyridinecarboxamide:

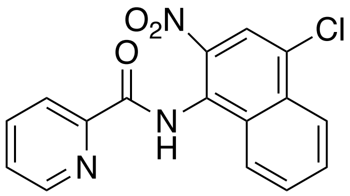

This compound was made according to the general procedure for nitration reaction described above starting from 142 mg (0.5 mmol) of substrate to yield a pale yellow crystalline solid (70 mg, 43 %). This yield can be increased by introducing a higher loading of TBN. ^{1}H NMR ($CDCl_3$, 400 MHz, 298 K); δ = 11.18 (br s, 1H), 8.81 (d, 1H, $^3J_{HH}$ = 4.7 Hz), 8.41 (d, 1H, $^3J_{HH}$ = 8.5 Hz), 8.33 (d, 1H, $^3J_{HH}$ = 7.8 Hz), 8.26 (s, 1H), 8.23 (d, 1H, $^3J_{HH}$ = 8.5 Hz), 8.00 (ddd, 1H, $^3J_{HH}$ = 7.5, $^3J_{HH}$ = 7.5, $^3J_{HH}$ = 1.7 Hz), 7.89-7.83 (m, 1H), 7.79-7.73 (m, 1H), 7.65-7.59 (m, 1H). ^{13}C{^{1}H} NMR ($CDCl_3$, 100 MHz, 298 K); δ = 163.41, 148.64, 140.98, 137.80, 133.14, 131.26, 130.65, 129.95, 128.54, 127.30, 126.82, 125.17, 123.06, 120.69. HR-MS (ESI, m/z); calcd. for $C_{16}H_{10}ClN_3O_3$+H = 328.0489; obtained = 328.0484 $[M+H]^+$. R_f = 0.83 (ethyl acetate/dichloromethane = 1/3).

N-(2-nitro-4-methoxy-1-naphthalenyl)-2-pyridinecarboxamide:

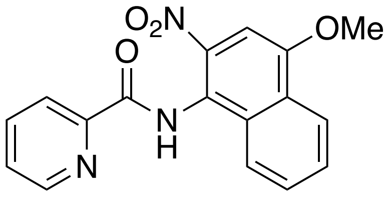

This compound was made according to the general procedure for nitration reaction described above starting from 141 mg, (0.5 mmol) of substrate to yield a pale yellow crystalline solid (160 mg, >99%). ^{1}H NMR ($CDCl_3$, 400 MHz, 298 K); δ = 10.89 (br s, 1H), 8.80, (d, 1H, $^3J_{HH}$ = 4.8 Hz), 8.42-8.37 (m, 1H), 8.33 (d, 1H, $^3J_{HH}$ = 7.7 Hz), 8.16-8.11 (m, 1H), 7.98 (ddd, 1H, $^3J_{HH}$ = 8.0, $^3J_{HH}$ = 8.0, $^4J_{HH}$ = 1.6 Hz), 7.75-7.68 (m, 2H), 7.63-7.56 (m, 1H), 7.44 (s, 1H), 4.15 (s, 3H). ^{13}C{^{1}H} NMR ($CDCl_3$, 100 MHz, 298 K); δ = 163.48, 154.70, 149.08, 148.56, 142.38, 137.65, 130.00, 128.90, 128.43, 128.08, 127.00, 126.06, 122.91, 122.65, 122.24, 98.71, 56.25. HR-MS (ESI, m/z); calcd. for $C_{17}H_{13}N_3O_4$+H = 324.0984; obtained = 324.0980 $[M+H]^+$. R_f = 0.78 (ethyl acetate/dichloromethane = 1/3).

N-(4-amino-1-naphthaleneyl)-2-pyridinecarboxamide:

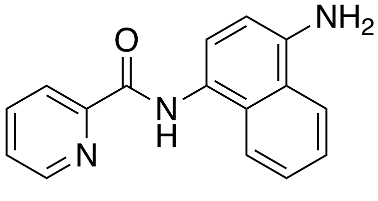

N-(4-nitro-1-naphthalenyl)-2-pyridinecarboxamide (97 mg, 0.33 mol) was dissolved in 10 mL ethyl acetate and Pd/C (50 mg, 50 wt%) were added to a sealed round bottom flask. The reaction was left under stirring at room temperature under an atmosphere of hydrogen (1 atm, balloon). When the reaction was completed, it was filtered through celite® and the celite® further washed with ethyl acetate until the filtrate ran clear. The combine organic filtrates were then removed under reduced pressure and the resulting residue was purified by column chromatography using ethyl acetate: dichloromethane (20:80) as eluent to yield a pale brown powder (87 mg, >99%). ^{1}H NMR (D_6-DMSO, 400 MHz, 298 K); δ = 10.42 (br s, 1H), 7.78 (d, 1H, $^3J_{HH}$ = 3.8 Hz), 8.17-8.04 (m, 3H), 7.77 (d, 1H, $^3J_{HH}$ = 8.1 Hz),

7.72-7.65 (m, 1H), 7.49-7.40 (m, 3H), 6.70 (d, 1H, $^{3}J_{HH}$ = 8.1 Hz), 5.78 (br s, 2H). $^{13}C\{^{1}H\}$ NMR ($CDCl_3$, 100 MHz, 298 K); δ = 162.58, 150.31, 148.14, 140.26, 137.67, 128.34, 126.37, 125.07, 124.17, 124.09, 122.50, 121.85, 121.69, 121.52, 109.61. HR-MS (ESI, m/z); calcd. for $C_{16}H_{13}N_3O$+H = 264.1137; obtained = 264.1138 $[M+H]^+$. R_f = 0.51 (ethyl acetate/dichloromethane = 1/3).

4-Nitro-1-aminonaphthalene:

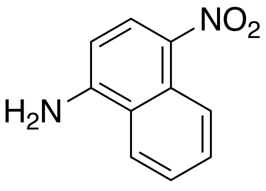

N-(4-nitro-1-naphthalenyl)-2-pyridinecarboxamide (80 mg, 0.27 mmol) was dissolved in ethanol (4.0 mL) in a 10 mL vial. Concentrated hydrochloric acid (2.0 mL) was added dropwise into the reaction mixture. After heating at 100 °C for 24 hours, the crude was concentrated under reduced pressure and cooled to 0 °C. Thereafter, 2M NaOH was added dropwise until the pH was alkaline. The solution was then extracted with ethyl acetate (3 x 50 mL), dried over magnesium sulfate and concentrated under reduced pressure. The resulting residue was purified by column chromatography using ethyl acetate: dichloromethane (20:80) as eluent to yield an orange amorphous solid (43 mg, 85%). ^{1}H NMR (D_6-DMSO, 400 MHz, 298 K); δ = 8.92 (d, 1H, $^{3}J_{HH}$ = 8.6 Hz), 8.40 (d, 1H, $^{3}J_{HH}$ = 9.0 Hz), 8.31 (d, 1H, $^{3}J_{HH}$ = 7.8 Hz), 7.78-7.72 (m, 1H), 7.60 (br s, 2H), 7.57-7.52 (m, 1H), 6.68 (d, 1H, $^{3}J_{HH}$ = 9.0 Hz). HR-MS (ESI, m/z); calcd. for $C_{10}H_8N_2O_2$+H = 189.0664; obtained = 189.0658 $[M+H]^+$. R_f = 0.67 (ethyl acetate/dichloromethane = 1/3). This data is consistent with the proposed structure and the previously reported characterisation data.[5]

1,4-diaminonaphthalene:

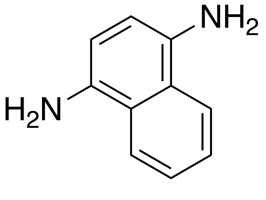

4-Nitro-1-aminonaphthalene (50 mg, 0.26 mmol) was dissolved in 8 mL ethyl acetate and Pd/C (16 mg, 50 wt%) in a sealed round bottom flask. The reaction was left under stirring at room temperature under an atmosphere of hydrogen (1 atm, balloon). When the reaction was completed, it was filtered through celite® and the celite® was further washed with ethyl acetate until the filtrate ran clear. The combined organic filtrates were then removed under reduced pressure, resulting in a dark purple solid (32 mg, 78%). ^{1}H NMR (D_6-DMSO, 400 MHz, 298 K); δ = 7.98 (d, 2H, $^{3}J_{HH}$ = 8.6 Hz), 7.38 (d, 2H, $^{3}J_{HH}$ = 8.2 Hz), 6.59 (d, 2H, $^{3}J_{HH}$ = 9.0 Hz), 4.84 (br s, 4H). HR-MS (ESI, m/z); calcd. for $C_{10}H_{10}N_2$+H = 159.0922; obtained = 159.0918 $[M+H]^+$. R_f = 0.33 (ethyl acetate/dichloromethane = 1/3). This data is consistent with the proposed structure and the previously reported characterisation data.[6]

N-(2-amino-4-chloro-1-naphthaleneyl)-2-pyridinecarboxamide:

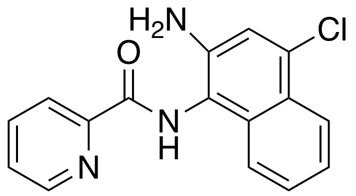

N-(2-nitro-4-chloro-1-naphthalenyl)-2-pyridinecarboxamide (147 mg, 0.45 mmol) was dissolved in 15 mL ethyl acetate and Pd/C added (100 mg, 50 wt%) to a sealed round bottom flask. The reaction was left under stirring at room temperature under an atmosphere of hydrogen (1 atm, balloon). When the reaction was completed, it was filtered through celite® and the celite® was further washed with ethyl acetate until the filtrate ran clear. The combined organic filtrates were then removed under reduced pressure, resulting in a pale green amorphous powder (120 mg, 90%). ^{1}H NMR (D_6-DMSO, 400 MHz, 298 K); δ = 10.11 (br s, 1H), 7.75 (d, 1H, $^{3}J_{HH}$ = 4.3 Hz), 8.10 (d, 1H, $^{3}J_{HH}$ = 7.6 Hz), 8.04 (dd, 1H, $^{3}J_{HH}$ = 7.4, $^{3}J_{HH}$ = 7.4 Hz), 7.94 (d, 1H, $^{3}J_{HH}$ = 8.3 Hz), 7.66 (dd, 1H, $^{3}J_{HH}$ = 6.4, $^{3}J_{HH}$ = 6.4 Hz), 7.53 (d, 1H, $^{3}J_{HH}$ = 8.5 Hz), 7.39 (dd, 1H, $^{3}J_{HH}$ = 7.7, $^{3}J_{HH}$ = 7.7 Hz), 7.32 (s, 1H), 7.30-7.23 (m, 1H), 5.45 (br s, 2H). ^{13}C{^{1}H} NMR (D_6-DMSO, 100 MHz, 298 K); δ = 163.65, 150.10, 148.47, 143.06, 137.77, 132.94, 129.74, 127.13, 126.68, 123.17, 122.34, 121.67, 118.48, 112.08. HR-MS (ESI, m/z); calcd. for $C_{16}H_{12}ClN_3O$+H = 298.0747; obtained = 298.0732 $[M+H]^+$. R_f = 0.54 (ethyl acetate/dichloromethane = 1/3).

[4] Original Spectra for New Products:

This section contains copies of the original ^{1}H, ^{13}C {^{1}H} and COSY NMR spectra obtained for all previously unreported products prepared in this study.

N-(4-nitro-1-naphthalenyl)-2-pyridinecarboxamide:

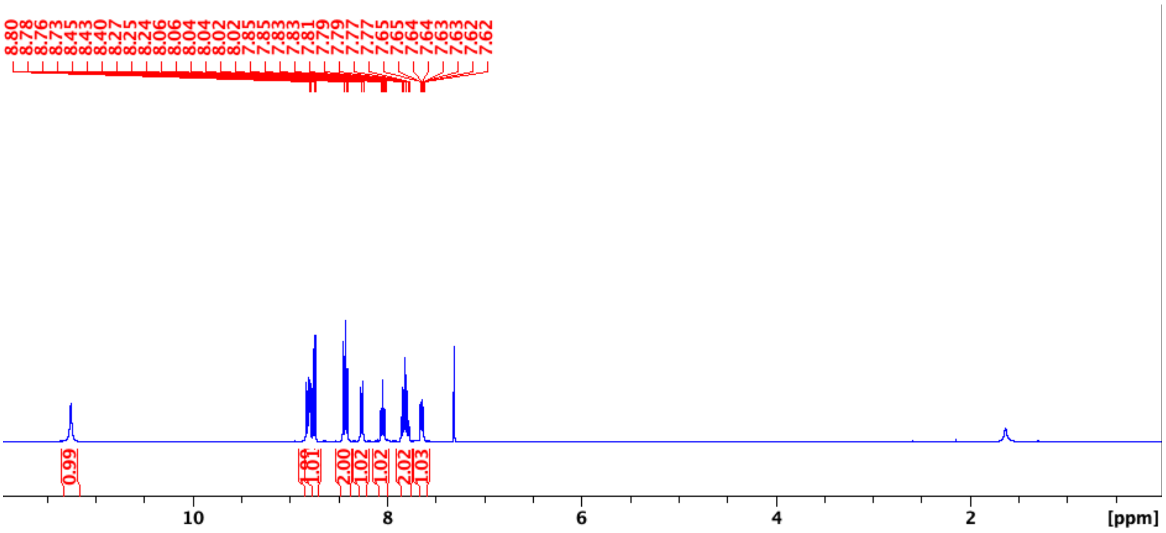

Figure S1: ^{1}H NMR spectrum in $CDCl_3$ at 298 K.

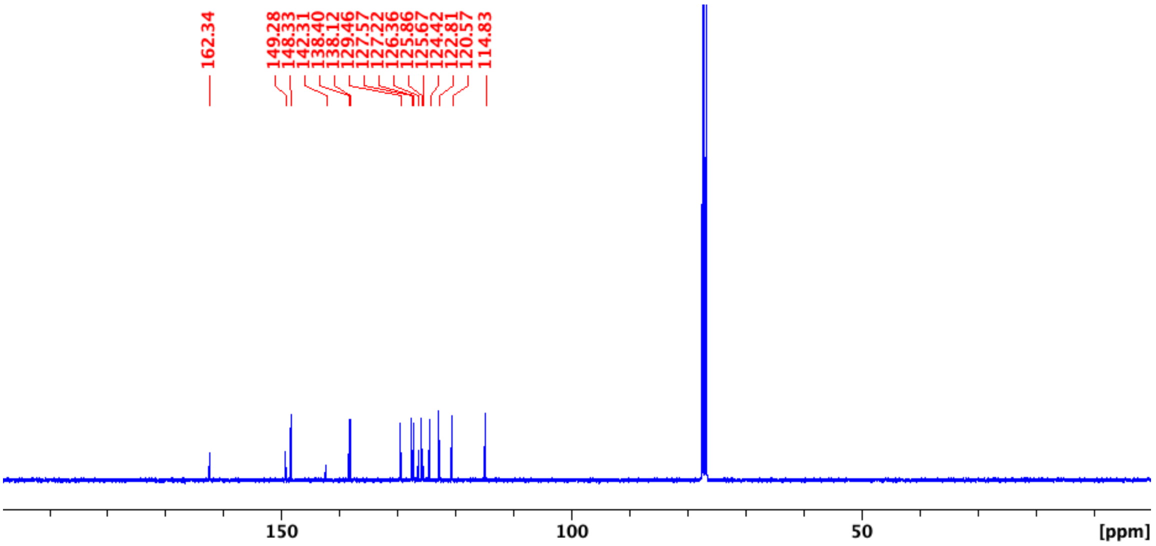

Figure S2: ^{13}C {^{1}H} NMR spectrum in $CDCl_3$ at 298 K.

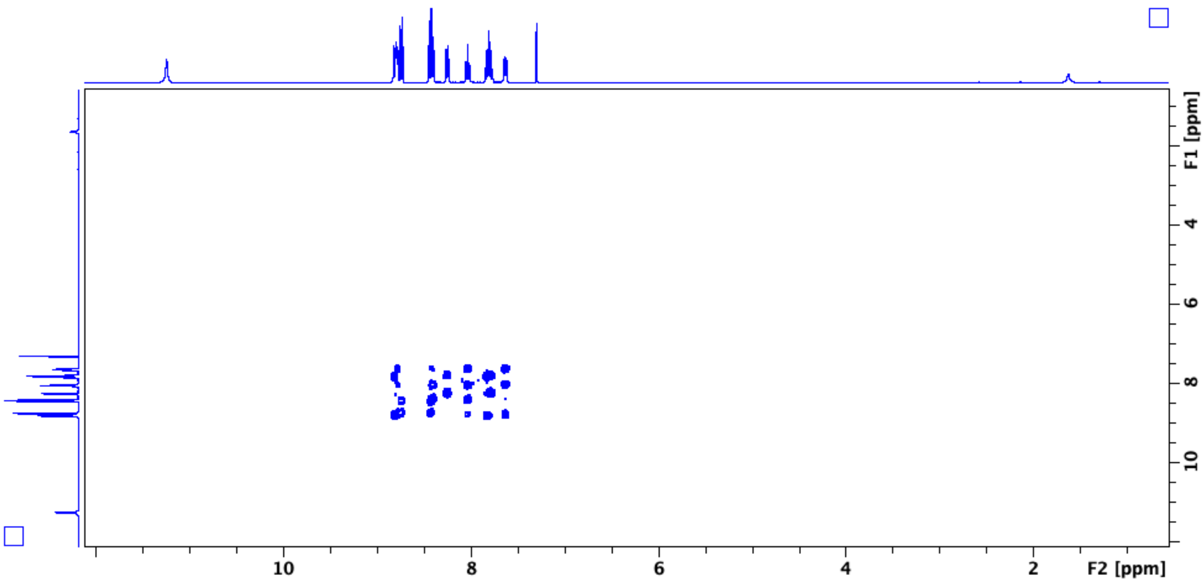

Figure S3: COSY NMR spectrum in $CDCl_3$ at 298 K.

<u>*N*-(2-nitro-4-chloro-1-naphthalenyl)-2-pyridinecarboxamide:</u>

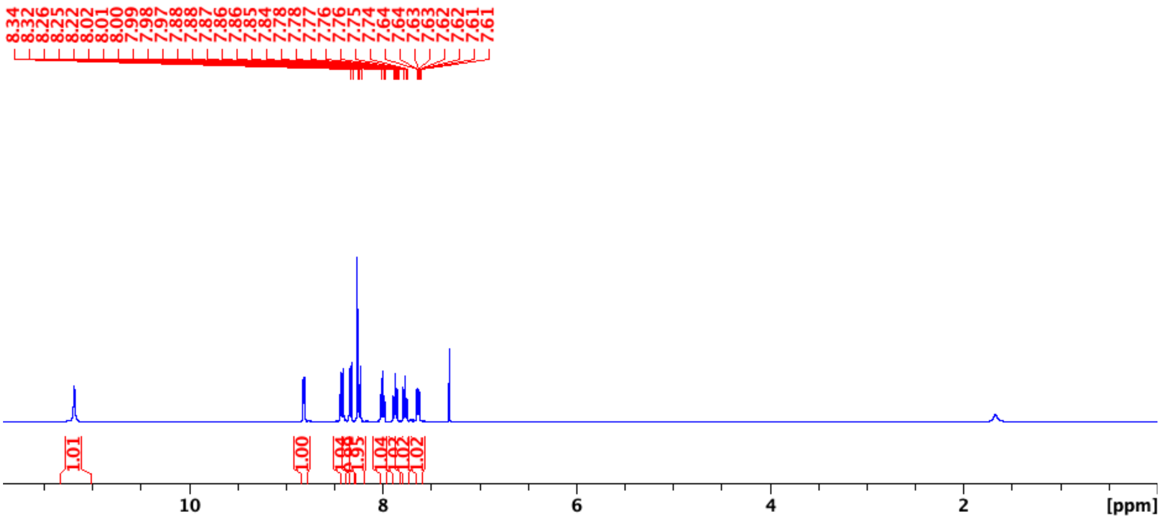

Figure S4: ^1H NMR spectrum in $CDCl_3$ at 298 K.

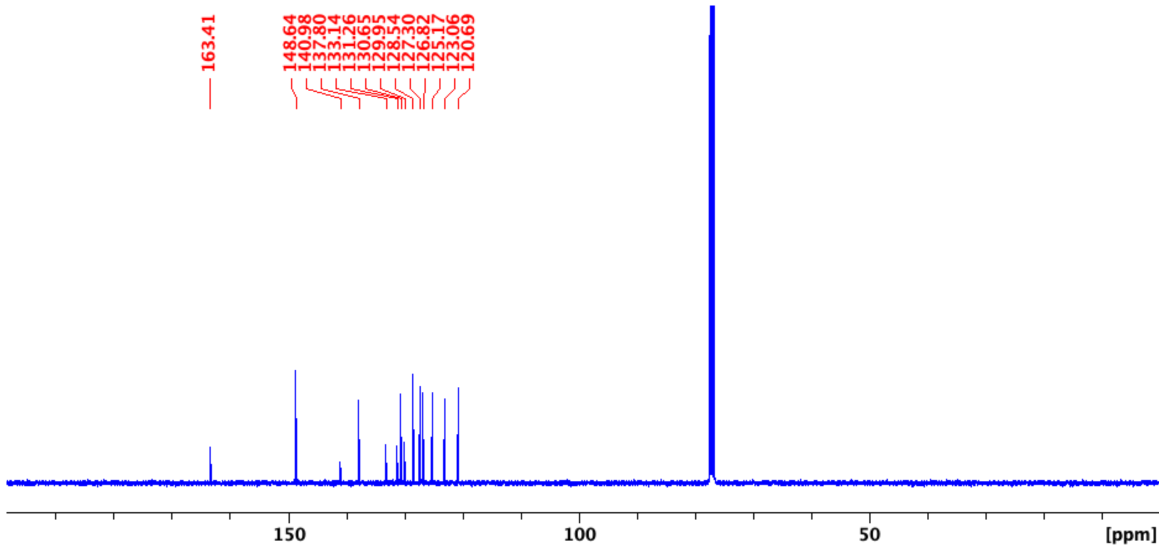

Figure S5: $^{13}C\ \{^{1}H\}$ NMR spectrum in $CDCl_3$ at 298 K.

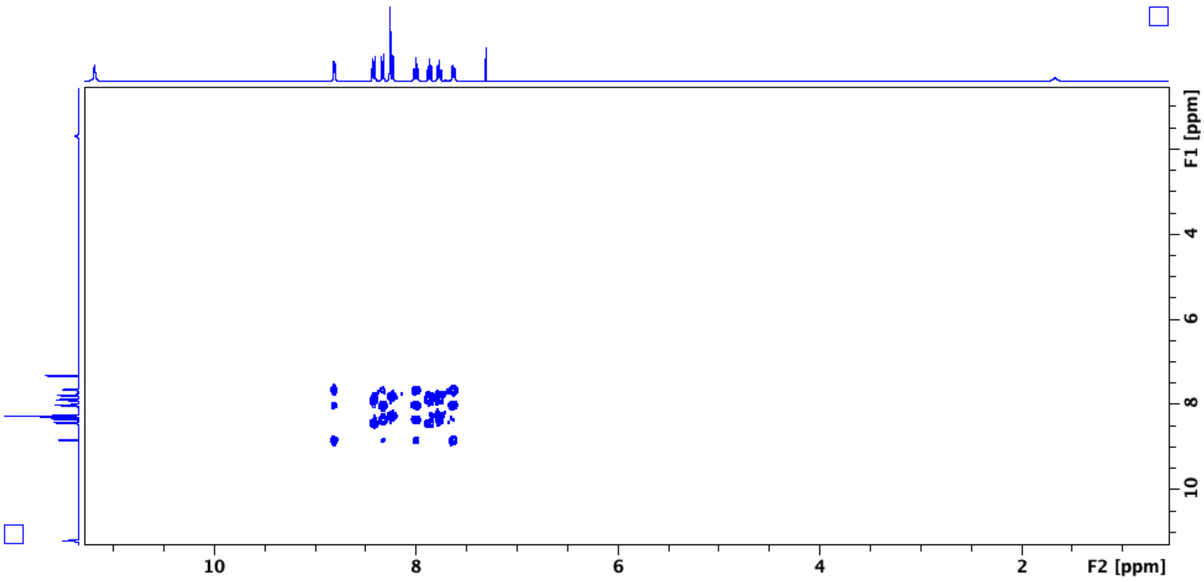

Figure S6: COSY NMR spectrum in $CDCl_3$ at 298 K.

<u>*N*-(2-nitro-4-methoxy-1-naphthalenyl)-2-pyridinecarboxamide:</u>

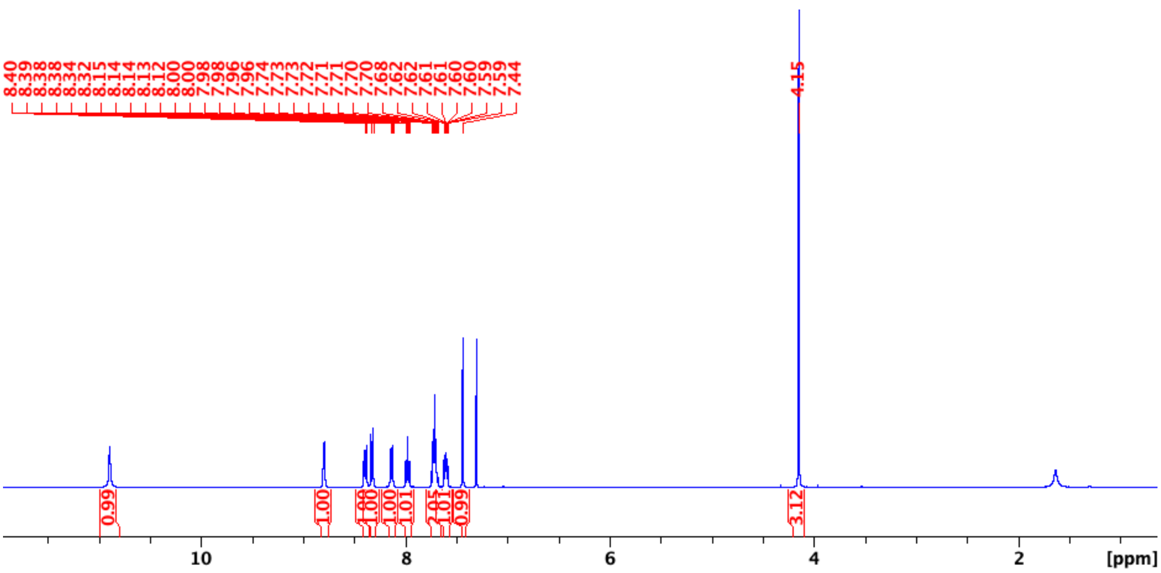

Figure S7: ^{1}H NMR spectrum in $CDCl_3$ at 298 K.

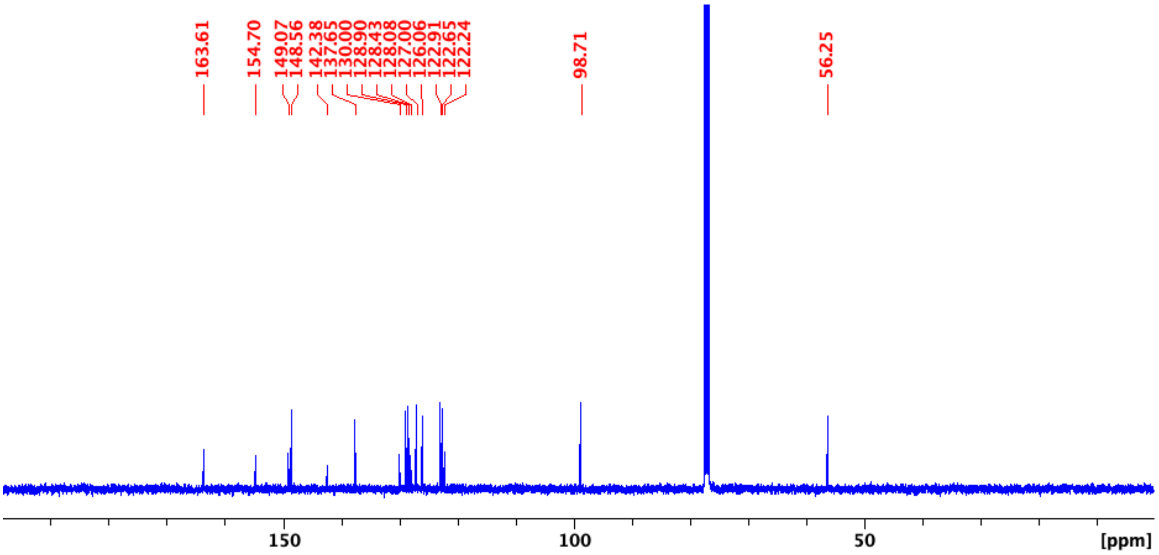

Figure S8: $^{13}C\ \{^{1}H\}$ NMR spectrum in $CDCl_3$ at 298 K.

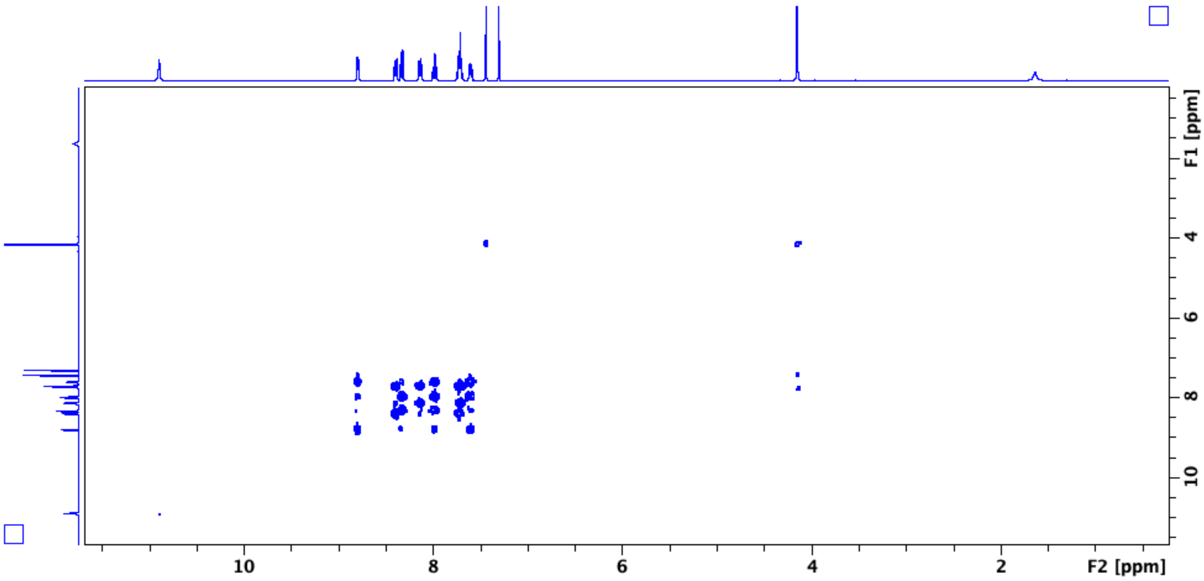

Figure S9: COSY NMR spectrum in $CDCl_3$ at 298 K.

<u>*N*-(2-amino-4-chloro-1-naphthaleneyl)-2-pyridinecarboxamide:</u>

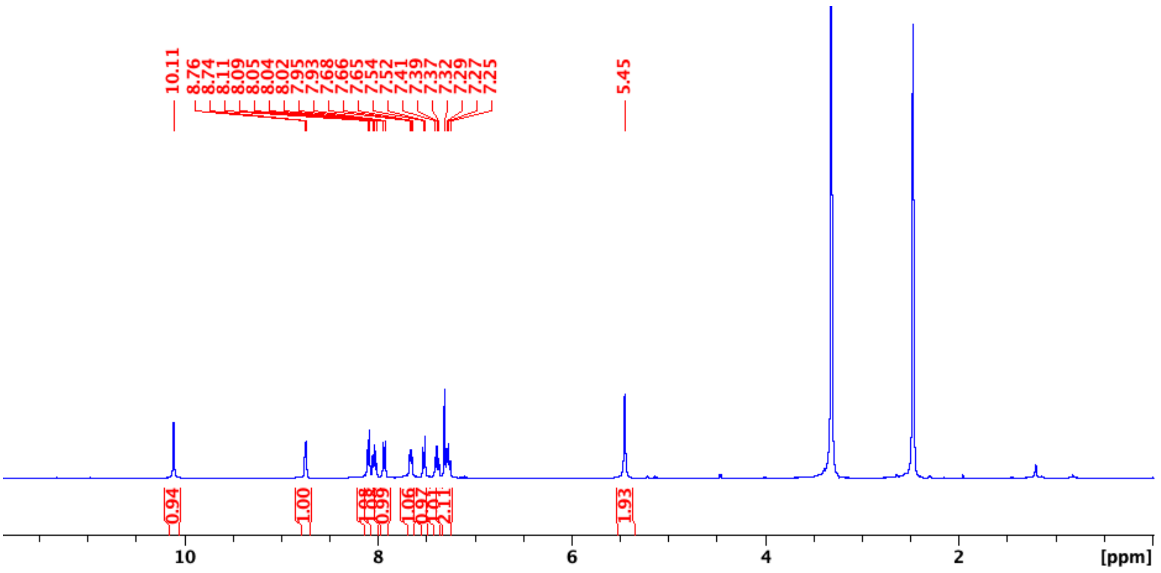

Figure S10: ^{1}H NMR spectrum in D_6-DMSO at 298 K.

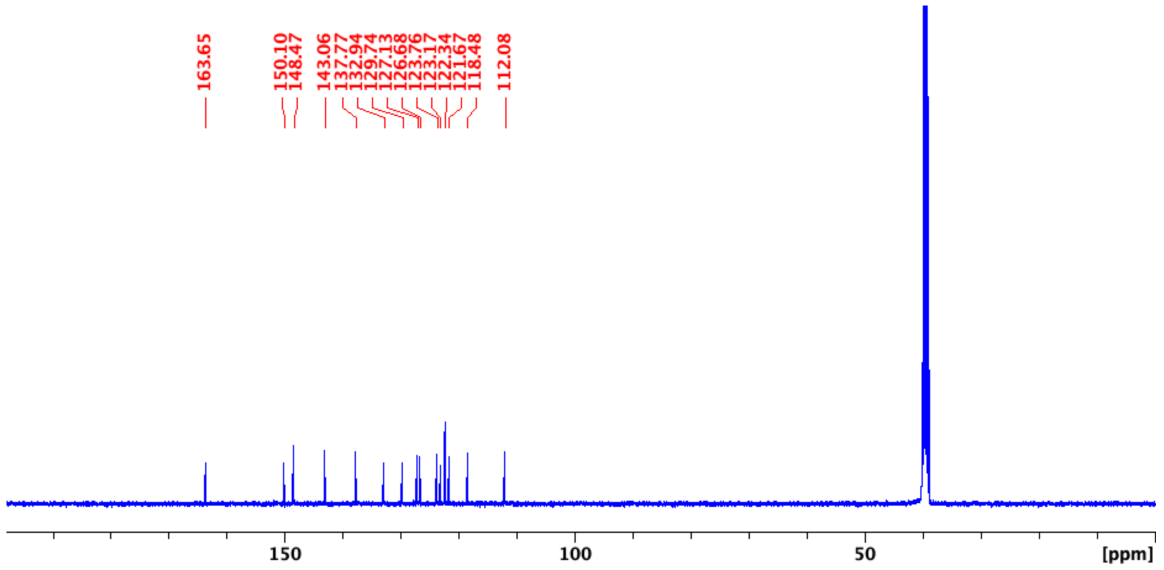

Figure S11: $^{13}C\ \{^{1}H\}$ NMR spectrum in D_6-DMSO at 298 K.

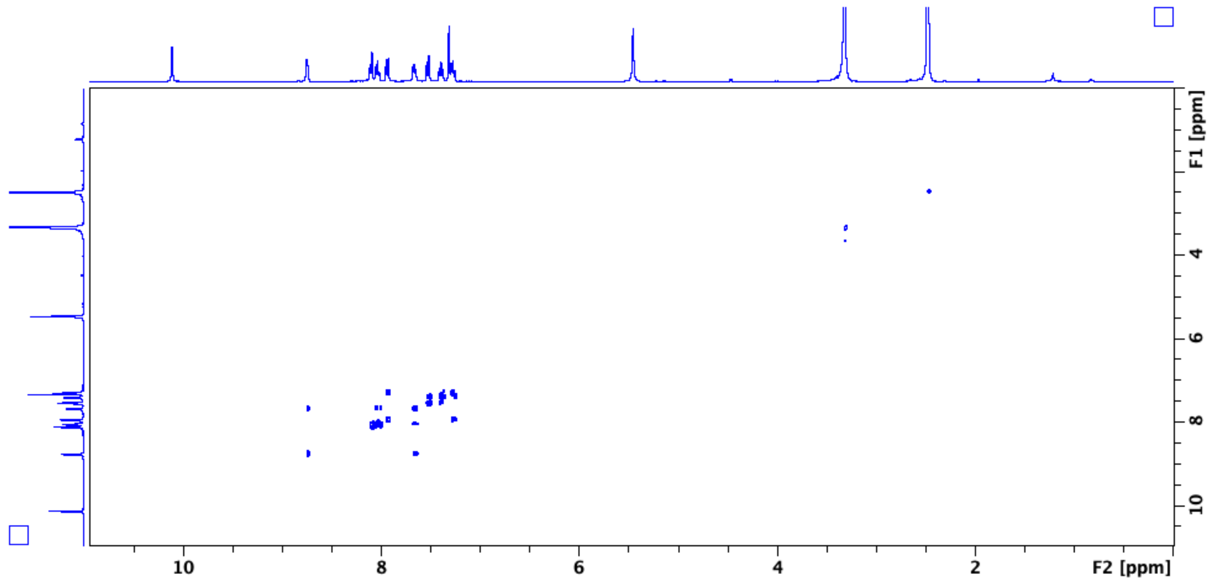

Figure S12: COSY NMR spectrum in D_6-DMSO at 298 K.

<u>*N*-(4-amino-1-naphthaleneyl)-2-pyridinecarboxamide:</u>

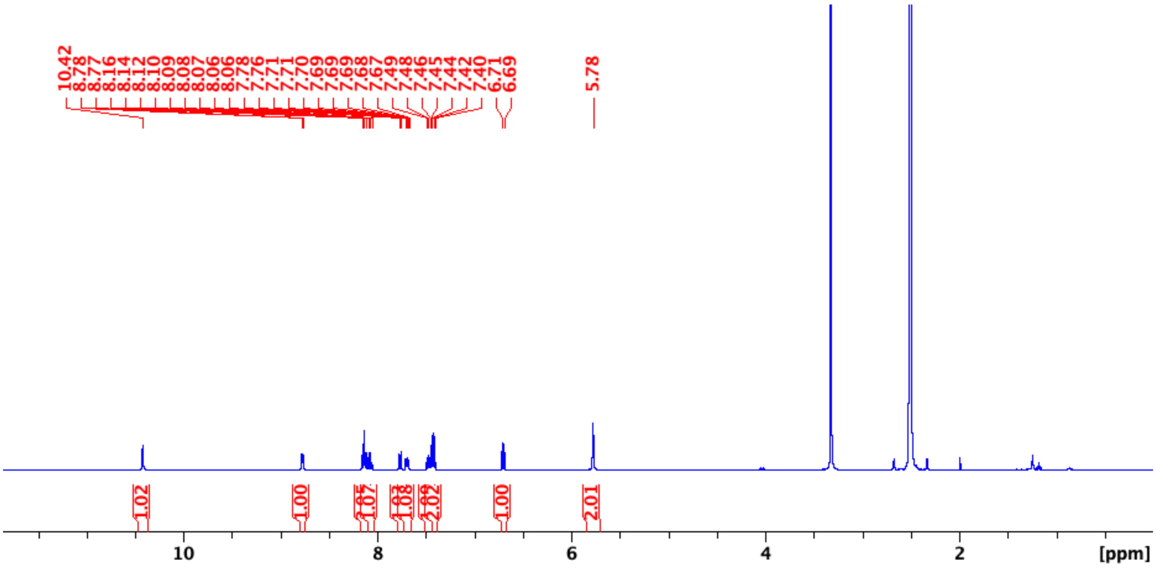

Figure S13: ^{1}H NMR spectrum in D_6-DMSO at 298 K. Note: poor spectra quality is obtained if a normal/high concentration sample is prepared.

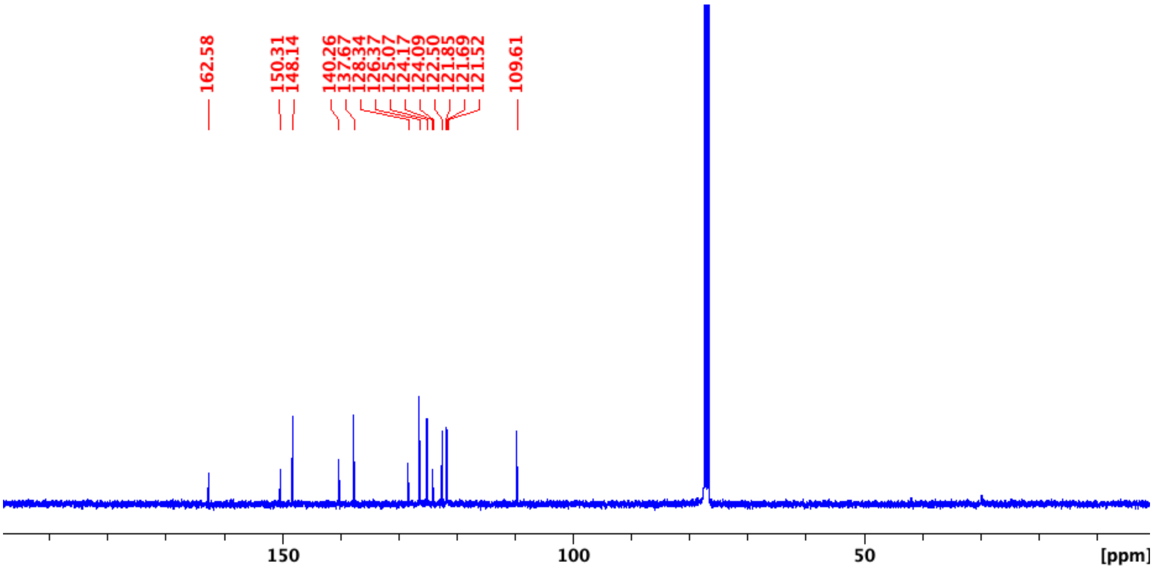

Figure S14: ^{13}C $\{^{1}H\}$ NMR spectrum in D_6-DMSO at 298 K. Note: poor spectra quality is obtained if a normal/high concentration sample is prepared.

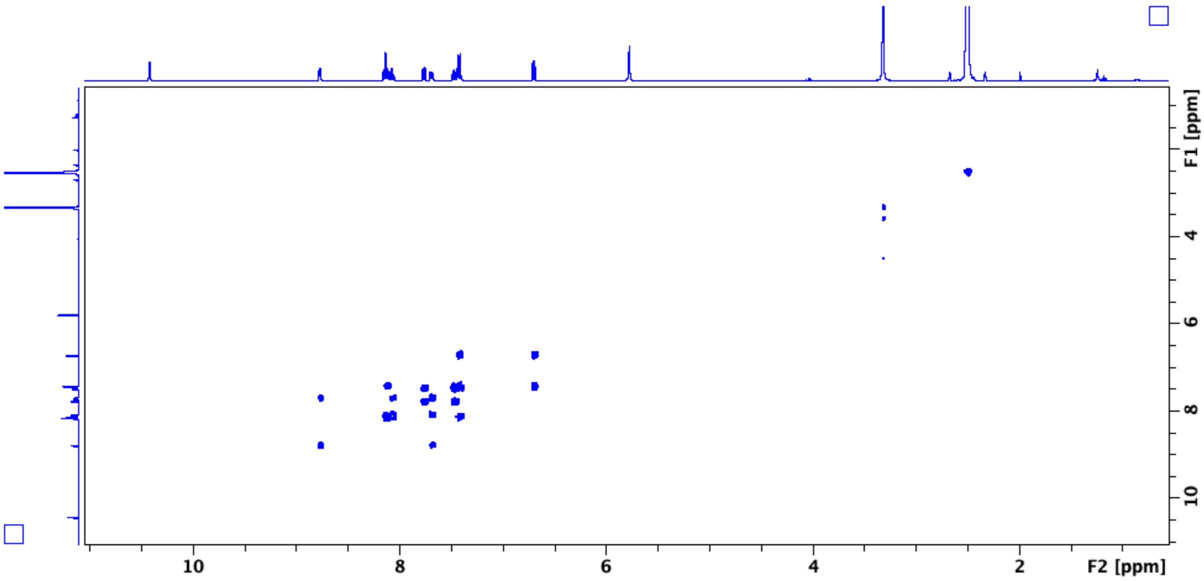

Figure S15: COSY NMR spectrum in D_6-DMSO at 298 K. Note: poor spectra quality is obtained if a normal/high concentration sample is prepared.

[5] Computational Details:

DFT calculations for optimisations and analytical frequencies were performed using the ORCA 3.03 computational software[7] at the RI-B97-D3/def2-TZVP level of theory[8-10] and single point energies and solvation corrections calculated at RIJCOSX-M06L/def2-TZVP.[10-12] Solvation correction was implemented with the COSMO[13] model for acetic acid (ε = 6.15). Graphical visualisation using Gabedit 2.4.8[14] and Avogadro 1.2.0[15] programs. Analytical frequencies were calculated for inclusion of the Zero Point Energy (ZPE) correction and entropic contributions to the free energy term (ΔG_{298K}), as well as confirming all intermediate were true with no imaginary modes and all transition states had the correct critical frequency of decomposition (imaginary mode). To obtain a more accurate description of the energetics of the reaction, and spin state energy gaps, open shell DLPNO-CCSD(T)/def2-TZVP[16] calculations where performed on all structures. The DLPNO-CCSD(T) calculations were implemented with ORCA 4.0.1. The DLPNO-CCSD(T) energies where corrected with the solvation and free energy terms from the previous RIJCOSX-M06L/def2-TZVP and RI-B97-D3/def2-TZVP respectively, giving the results discussed in the text. Due the size of system of interest the *normal*-PNO settings were used, which has been shown to be within 2-4 kcal mol^{-1} of canonical CCSD(T) calculations,[17] but with a significant reduction in cost. Spin densities, when discussed in the text, have been calculated with DLPNO-CCSD/def2-TZVP with ORCA 4.1.1. At the time of completion, the triples correction to the density was not available for open-shell complexes.

QTAIM[18] and Bond Order[19-21] analysis was performed using the Multiwfn software package.

Table S1: T1 diagnostic from DLPNO-CCSD(T)/def2-tzvp calculations.

Aminoquinoline Species	T1 diagnostic		Napththylpicolinamide Species	T1 diagnostic
^{1}Int 1	0.015762184		^{1}Int 1	0.016094578
^{3}Int 1	0.018394787		^{3}Int 1	0.018697355
^{5}Int 1	0.020279767		^{5}Int 1	0.021945446
^{2}TS 1-2$_{A}$	0.022041169		^{2}TS 1-2$_{A}$	0.018205083
^{4}TS 1-2$_{A}$	0.026603031		^{4}TS 1-2$_{A}$	0.029677810
^{2}TS 1-2$_{B}$	0.021366149		^{2}TS 1-2$_{B}$	0.018295610
^{4}TS 1-2$_{B}$	0.0264004307		^{4}TS 1-2$_{B}$	0.032121043
^{2}Int 2$_{A}$	0.021050045		^{2}Int 2$_{A}$	0.017458373
^{4}Int 2$_{A}$	0.026223466		^{4}Int 2$_{A}$	0.026148268
^{2}Int 2$_{B}$	0.020576641		^{2}Int 2$_{B}$	0.019892107
^{4}Int 2$_{B}$	0.025391595		^{4}Int 2$_{B}$	0.023391163
^{5}Int 3$_{A}$	0.015753129			
^{5}Int 3$_{B}$	0.015701114			
^{5}TS 3-4$_{A}$	0.016493126			
^{5}TS 3-4$_{B}$	0.015915674			
^{4}Int 4$_{A}$	0.018852135			
^{4}Int 4$_{B}$	0.018705474			

Table S2. TDDFT calculated first transition energies for ***^{1}Int 1·L^1*** *and* ***^{1}Int 1·L^2****. Corresponding to a HOMO → LUMO ligand to metal based single electron transfer.*

Complex	Methodology	Transition Energy	
		**eV**	**kcal mol^{-1}**
^{1}Int 1·L^1	PBE0/def2-tzvp	1.605	37.01
	$PBE0_{solv}$ /def2-tzvp	1.600	36.90
	PBE0/def2-tzvpp	1.610	37.13
	M06L/def2-tzvp	1.652	38.10
	$M06L_{solv}$/def2-tzvp	1.410	32.52
	M06L/def2-tzvpp	1.667	38.44
	CAM-B3LYP/ def2-tzvp	1.728	39.85
	$CAM\text{-}B3LYP_{solv}$/ def2-tzvp	1.727	39.83
	CAM-B3LYP/ def2-tzvpp	1.731	39.92
	B2PLYP/def2-tzvp	1.891	43.61
	$B2PLYP_{solv}$/def2-tzvp	1.898	43.77
	B2PLYP/def2-tzvpp	1.891	43.61
^{1}Int 1·L^2	PBE0/def2-tzvp	1.633	37.66

All structures optimised with B97-D3/def2-tzvp. Solvent corrected excitations implemented with COSMO, epsilon = 6.15 to represent acetic acid.

[6] Coordinates from Computational Study:

Optimised Structure Coordinates

NO_2

N 0.002205 -0.035226 0.000009
O 1.165097 0.126757 0.325874
O -0.627154 -0.99178 -0.320384

$^{2}Co(OAc)_2$

C -1.287087 2.31851 -1.80740
O -0.16756 2.215896 -1.184557
O -1.677443 1.25517 -2.40055
C 1.141917 -1.557285 -1.737062
O 0.022312 -1.454611 -2.35976
O 1.532315 -0.494026 -1.14380
Co -0.07259 0.380612 -1.772135
C -2.068539 3.591158 -1.85366
H -3.10885 3.38672 -2.113962
H -1.63562 4.24715 -2.619068
H -2.00315 4.108668 -0.892495
C 1.923536 -2.82984 -1.691213
H 2.96358 -2.625452 -1.429818
H 1.859122 -3.346466 -2.652935
H 1.490026 -3.48667 -0.9268713

$^{4}Co(OAc)_2$

C -1.29711 2.41744 -1.845728
O -0.02103 2.497257 -1.778285
O -1.821371 1.251682 -1.878301
C 1.258987 -1.591682 -1.717704
O 0.885059 -1.009254 -0.641621
O 0.988301 -1.020588 -2.830270
Co 0.067987 0.467200 -1.782478
C -2.152400 3.649688 -1.863709
H -2.995138 3.512159 -2.545688
H -1.56309 4.524981 -2.1426753
H -2.559642 3.804709 -0.85776
C 1.963737 -2.915401 -1.679451
H 2.699272 -2.976911 -2.485287
H 1.220769 -3.705457 -1.841990
H 2.433688 -3.073636 -0.707042

OAc^-

C -0.30686 2.416736 0.52412
O -0.8199 2.087305 -0.577719
O 0.166719 1.686434 1.433454
C -0.28923 3.964843 0.813554
H 0.445182 4.225264 1.587049
H -1.284029 4.271234 1.170707
H -0.086299 4.533785 -0.103837

AcOH

H -0.690592 0.879204 0.303412
C -1.283972 -0.237501 1.700842
O -1.322363 0.958678 1.040037
O -0.569224 -1.149677 1.355046
C -2.226684 -0.242561 2.876691
H -1.930913 0.532267 3.592129
H -2.203845 -1.220824 3.356961
H -3.24353 -0.0081783 2.545699

Aminoquinoline Structures

^{1}Int 1

C 0.624903 0.281621 1.371934
C 1.660483 0.650363 2.240360
C 1.372021 1.149934 3.525007
C 0.081488 1.288752 4.000019
H -2.625840 1.370134 4.522451
C -2.363455 0.996903 3.535723
C -3.342942 0.598688 2.650554
C -2.978845 0.112219 1.382012
N -1.703854 0.031478 1.014770
C -0.706227 0.416257 1.864258
C -0.999160 0.916922 3.167672
Co -1.036493 -0.754645 -0.57359
H -3.714361 -0.230423 0.664024
H -4.395209 0.644890 2.913232
H 2.207985 1.427375 4.162626
H 2.685896 0.543036 1.918136
N 0.699875 -0.130282 0.049775
C 1.922611 -0.405735 -0.551266
O 2.950532 -0.638072 0.089200
C -2.713388 0.060940 -1.998243
O -2.863811 -1.049118 -1.38872
O -1.656639 0.724300 -1.724504
C 1.981330 -0.340673 -2.043685
C 2.855248 -1.200574 -2.722084
C 2.979704 -1.127218 -4.105923
C 2.254174 -0.171971 -4.824337
C 1.405979 0.706733 -4.149374
C 1.266503 0.621112 -2.764803
H 3.430080 -1.921970 -2.148371
H 3.646958 -1.808697 -4.628229
H 2.357789 -0.109022 -5.905352
H 0.852734 1.463103 -4.700774
H 0.606092 1.300345 -2.239842
C -0.290671 -2.911977 -0.98968
O -0.325358 -2.037068 -1.91355
O -0.688669 -2.521167 0.164197
H -0.116879 1.666959 4.998519
C 0.223174 -4.295855 -1.220021
H -0.229302 -4.995036 -0.51378
H 1.308464 -4.291663 -1.061114
H 0.027899 -4.601971 -2.250508
C -3.701697 0.553989 -3.009924

H -3.705259 1.646306 -3.038932
H -4.699369 0.168449 -2.787129
H -3.402191 0.183637 -3.997510

[1]Int 1$_{ES}$

C	0.595546	0.195976	1.222304
C	1.717088	0.694750	1.878230
C	1.565294	1.456535	3.042661
C	0.331087	1.735762	3.579098
H	-2.319272	2.006225	4.318012
C	-2.149472	1.432435	3.413359
C	-3.198418	0.882055	2.724411
C	-2.949731	0.148299	1.556367
N	-1.729938	-0.034525	1.098804
C	-0.683241	0.483497	1.776855
C	-0.834036	1.240957	2.949747
Co	-1.160417	-1.014973	-0.477233
H	-3.746649	-0.302103	0.976961
H	-4.218681	1.004553	3.064069
H	2.455482	1.829175	3.535552
H	2.707096	0.475546	1.502221
N	0.544221	-0.531251	0.078366
C	1.770494	-0.955952	-0.500468
O	2.513419	-1.664485	0.135464
C	-2.929529	-0.198306	-1.893162
O	-2.990218	-1.213525	-1.127048
O	-1.903567	0.511565	-1.886295
C	2.091509	-0.470372	-1.860771
C	3.251083	-0.958155	-2.463583
C	3.639021	-0.489092	-3.703877
C	2.875674	0.477601	-4.348187
C	1.720590	0.962303	-3.753879
C	1.322226	0.488391	-2.513763
H	3.835248	-1.702972	-1.936846
H	4.539367	-0.871876	-4.170201
H	3.182931	0.850930	-5.318520
H	1.120145	1.709741	-4.258924
H	0.405142	0.851732	-2.069308
C	-0.680743	-3.263173	-1.115450
O	-0.522835	-2.210380	-1.825508
O	-1.093672	-3.150545	0.053717
H	0.239383	2.320003	4.486947
C	-0.331842	-4.590261	-1.712403
H	-0.909377	-5.379589	-1.234839
H	0.729316	-4.781696	-1.533630
H	-0.500568	-4.581750	-2.788659
C	-4.082385	0.102427	-2.799190
H	-4.106761	1.162795	-3.043964
H	-5.022910	-0.214658	-2.349846
H	-3.944796	-0.462304	-3.724384

[3]Int 1

C 0.502677 0.497976 2.276910
C 1.305199 0.798427 3.380815
C 0.717969 0.981906 4.648785
C -0.644827 0.869949 4.855996
H -3.400088 0.529564 4.797909
C -2.899843 0.406579 3.840368
C -3.617425 0.093875 2.702519
C -2.952920 -0.054438 1.471684
N -1.634943 0.093808 1.372127
C -0.900689 0.386353 2.486235
C -1.495437 0.563618 3.762449
Co -0.530901 -0.124670 -0.189279
H -3.477531 -0.299776 0.555013
H -4.693984 -0.040671 2.736918
H 1.368043 1.213029 5.488564
H 2.379246 0.874066 3.265412
N 0.880931 0.346074 0.956245
C 2.261529 0.281516 0.615197
O 3.038642 -0.381533 1.287813
C -2.113310 0.989389 -1.737156
O -2.103075 -0.255097 -1.406110
O -1.308660 1.786092 -1.193909
C 2.691036 1.064656 -0.569968
C 3.914112 0.740825 -1.176251
C 4.364492 1.467445 -2.272001
C 3.605865 2.535320 -2.761610
C 2.397231 2.871807 -2.150800
C 1.937389 2.139001 -1.059680
H 4.490344 -0.088725 -0.778096
H 5.305054 1.204128 -2.748334
H 3.958214 3.102488 -3.619752
H 1.805577 3.701208 -2.528645
H 0.988888 2.391019 -0.601738
C 0.540617 -1.945607 -1.376485
O 0.627376 -0.692289 -1.664814
O -0.153646 -2.298934 -0.386531
H -1.073899 1.006032 5.844619
C 1.309599 -2.930749 -2.214392
H 0.879505 -3.929337 -2.113900
H 2.345827 -2.955116 -1.856645
H 1.321235 -2.616863 -3.261400
C -3.084354 1.445320 -2.797906
H -3.401026 2.472510 -2.602466
H -3.947439 0.778601 -2.854801
H -2.570078 1.427032 -3.766273

[5]Int 1

C 0.625487 0.424924 1.259340
C 1.659757 0.995499 2.013366
C 1.408752 1.538890 3.285733
C 0.147217 1.526515 3.851756
H -2.490443 1.272462 4.606158
C -2.260825 0.874900 3.620603
C -3.245818 0.286337 2.851721
C -2.913771 -0.222880 1.583238
N -1.680451 -0.143797 1.103034
C -0.688569 0.416776 1.839458
C -0.934971 0.956762 3.133938
Co -0.983384 -0.802547 -0.735316
H -3.647048 -0.703280 0.941917
H -4.267483 0.205385 3.209037
H 2.238567 1.974713 3.835644

H 2.665629 1.006705 1.613079
N 0.721474 -0.089740 -0.010060
C 1.973503 -0.463567 -0.540797
O 2.863441 -0.936463 0.157816
C -2.861215 0.061644 -2.144138
O -3.120960 -1.044077 -1.620949
O -1.733088 0.649074 -1.882894
C 2.130037 -0.289237 -2.012088
C 3.099122 -1.052941 -2.678399
C 3.295546 -0.896152 -4.046060
C 2.538336 0.038003 -4.760057
C 1.583404 0.811432 -4.099252
C 1.376083 0.647670 -2.730792
H 3.683351 -1.768228 -2.107863
H 4.038524 -1.500591 -4.560182
H 2.694850 0.161345 -5.829119
H 0.996052 1.542223 -4.648854
H 0.629894 1.246755 -2.222059
C -0.546008 -3.233565 -0.913430
O -0.229246 -2.390447 -1.819567
O -1.106459 -2.811479 0.142533
H -0.028090 1.942188 4.839799
C -0.219502 -4.690706 -1.094029
H -0.892347 -5.310589 -0.498612
H 0.807205 -4.861510 -0.749136
H -0.272809 -4.963985 -2.150603
C -3.812046 0.757524 -3.084739
H -4.031544 1.763269 -2.712456
H -4.734951 0.185437 -3.190215
H -3.332892 0.870658 -4.063300

^{2}TS 1-2$_{A}$

C 0.548513 0.460227 2.116166
C 1.444311 0.772271 3.185769
C 0.953532 1.086149 4.423520
C -0.458123 1.219383 4.684705
H -3.223368 0.865534 4.791205
C -2.775328 0.635712 3.829168
C -3.559481 0.240799 2.749130
C -2.965775 -0.022098 1.511870
N -1.641260 0.079542 1.334919
C -0.864483 0.445927 2.391568
C -1.388487 0.753238 3.655250
Co -0.614849 -0.271241 -0.248330
H -3.536823 -0.322503 0.640316
H -4.635228 0.137682 2.850922
H 1.644372 1.300222 5.233546
H 2.514130 0.718294 3.013675
N 0.845544 0.144015 0.857745
C 2.223314 0.009983 0.432259
O 2.892528 -0.900206 0.881521
C -2.182101 0.851423 -1.790863
O -2.208932 -0.378463 -1.404323
O -1.332841 1.643163 -1.313247
N -0.794015 3.154333 4.647559
O -1.800497 3.493344 5.229633
O -0.009690 3.831444 4.031557
C 2.698255 1.008951 -0.546493
C 3.983877 0.846406 -1.089421
C 4.471864 1.765016 -2.010216
C 3.683080 2.854700 -2.394522
C 2.407513 3.022762 -1.853832
C 1.912504 2.105041 -0.931229
H 4.577728 -0.008855 -0.782033
H 5.463254 1.634091 -2.434812
H 4.063552 3.570529 -3.119432
H 1.789919 3.863118 -2.157977
H 0.910774 2.226348 -0.538105
C 0.441804 -2.125740 -1.415254
O 0.524725 -0.871649 -1.716458
O -0.241511 -2.475850 -0.420811
H -0.795255 1.127206 5.714204
C 1.210878 -3.112953 -2.252634
H 0.774017 -4.109310 -2.158825
H 2.243434 -3.145717 -1.885163
H 1.232405 -2.796960 -3.298879
C -3.172502 1.291205 -2.841790
H -3.407713 2.351033 -2.719861
H -4.081104 0.686106 -2.803124
H -2.713119 1.153528 -3.828131

^{2}TS 1-2$_{B}$

C 0.529681 0.546874 2.034939
C 1.446939 0.993175 3.068759
C 0.894218 1.132545 4.412028
C -0.436623 1.053955 4.662934
H -3.215626 0.824594 4.756497
C -2.771286 0.639046 3.782165
C -3.565190 0.285152 2.692958
C -2.978481 0.034737 1.450828
N -1.653452 0.138036 1.269658
C -0.870764 0.490970 2.326150
C -1.383207 0.744595 3.612951
Co -0.630513 -0.256380 -0.304983
H -3.552964 -0.259833 0.579818
H -4.641787 0.191441 2.795926
H 1.592352 1.351211 5.214376
H 2.477294 0.661777 2.991804
N 0.828753 0.230031 0.778147
C 2.209271 0.121376 0.348995
O 2.909897 -0.733536 0.858286
C -2.216855 0.724496 -1.920826
O -2.229617 -0.471431 -1.438848
O -1.372625 1.556957 -1.509176
N 1.876228 2.828597 2.616342
O 1.118864 3.390720 1.863894
O 2.858000 3.259194 3.173958
C 2.650022 1.064689 -0.695585
C 3.943968 0.908747 -1.222151
C 4.405537 1.777709 -2.202059

C 3.582676 2.812137 -2.660882
C 2.300255 2.974907 -2.135255
C 1.830589 2.105735 -1.154826
H 4.564861 0.097990 -0.853726
H 5.402981 1.651730 -2.613830
H 3.943426 3.490234 -3.430775
H 1.658312 3.774471 -2.493852
H 0.827973 2.227919 -0.766020
C 0.451243 -2.156870 -1.372568
O 0.515952 -0.921226 -1.743793
O -0.226106 -2.460138 -0.358417
H -0.815108 1.198816 5.671551
C 1.230068 -3.178159 -2.158348
H 0.894066 -4.186007 -1.907641
H 2.292005 -3.080788 -1.902638
H 1.125026 -2.993670 -3.231719
C -3.216001 1.071122 -2.997807
H -3.433392 2.141415 -2.982833
H -4.132851 0.488006 -2.883820
H -2.775382 0.822116 -3.970598

^{4}TS 1-2$_{A}$

C 0.592516 0.572458 1.803907
C 1.414861 0.949366 2.921230
C 0.893739 1.045873 4.178462
C -0.508476 0.857213 4.444057
H -3.098452 -0.103613 4.547269
C -2.648149 -0.146614 3.559281
C -3.368970 -0.622291 2.468565
C -2.764987 -0.645613 1.208623
N -1.509430 -0.232448 1.025831
C -0.792469 0.219064 2.076260
C -1.330678 0.294728 3.376084
Co -0.454574 -0.153808 -0.768044
H -3.281262 -0.999623 0.320946
H -4.389484 -0.972089 2.583027
H 1.537471 1.331394 5.005334
H 2.468193 1.137535 2.747764
N 0.968520 0.496160 0.538740
C 2.349017 0.556687 0.166983
O 3.153709 -0.201883 0.683105
C -2.197675 0.989276 -2.129619
O -2.287836 -0.262827 -2.106929
O -1.282038 1.580714 -1.434644
N -1.239631 2.693506 4.551341
O -2.294371 2.767040 5.140167
O -0.610572 3.562514 4.004080
C 2.689190 1.524285 -0.902258
C 3.953170 1.430350 -1.506917
C 4.316108 2.324961 -2.506223
C 3.424761 3.326295 -2.905901
C 2.171413 3.430152 -2.300901
C 1.800760 2.532526 -1.302171
H 4.628341 0.643289 -1.185812
H 5.290258 2.243109 -2.980656
H 3.707566 4.023016 -3.691519
H 1.476011 4.205289 -2.611289
H 0.819577 2.605837 -0.849571
C 0.605868 -2.230568 -1.577144
O 0.903054 -1.070048 -2.027529
O -0.256231 -2.331186 -0.653889
H -0.809903 0.623711 5.461868
C 1.285376 -3.454775 -2.131538
H 1.844293 -3.948969 -1.329482
H 1.964158 -3.189002 -2.943899
H 0.528136 -4.160444 -2.488494
C -3.122513 1.841452 -2.962832
H -3.433895 2.726531 -2.400781
H -3.991486 1.265611 -3.286357
H -2.576803 2.187873 -3.848136

^{4}TS 1-2$_{B}$

C 0.551623 0.654472 2.032185
C 1.373397 1.133750 3.145148
C 0.874945 0.929826 4.492192
C -0.410741 0.573750 4.728351
H -3.075559 -0.128366 4.825410
C -2.659317 -0.083848 3.821815
C -3.438431 -0.411711 2.716073
C -2.865636 -0.375336 1.442176
N -1.595171 -0.010739 1.255171
C -0.830858 0.327767 2.315169
C -1.319861 0.290058 3.642480
Co -0.531743 -0.047260 -0.529137
H -3.417876 -0.645857 0.546849
H -4.475929 -0.708008 2.831138
H 1.549872 1.130784 5.318106
H 2.445808 1.073074 2.994350
N 0.932755 0.554333 0.773460
C 2.316449 0.534585 0.396474
O 3.102105 -0.171292 1.009529
C -2.211986 1.116203 -1.952240
O -2.348138 -0.130595 -1.890324
O -1.285501 1.695849 -1.263130
N 1.279526 3.037615 2.884445
O 1.768286 3.413363 1.846617
O 0.748208 3.679856 3.755744
C 2.675694 1.316991 -0.807152
C 3.896131 1.035486 -1.440369
C 4.274729 1.746891 -2.572161
C 3.444088 2.752905 -3.075294
C 2.236097 3.045119 -2.440804
C 1.849167 2.329474 -1.311002
H 4.524542 0.248042 -1.036368
H 5.214011 1.517660 -3.068078
H 3.739575 3.306923 -3.962817
H 1.590320 3.828890 -2.827442
H 0.908429 2.559430 -0.827227
C 0.369047 -2.250643 -1.200490
O 0.745291 -1.144630 -1.722318

O -0.499110 -2.235621 -0.278181
H -0.778407 0.472307 5.746456
C -3.099340 1.976104 -2.817758
H -3.534252 2.782866 -2.218821
H -3.888669 1.378230 -3.276341
H -2.491706 2.443320 -3.600548
C 0.990792 -3.542588 -1.661402
H 0.314353 -4.380246 -1.476473
H 1.912541 -3.707233 -1.091326
H 1.252853 -3.482916 -2.720506

^{2}Int 2_A

C 0.549815 0.494570 2.082228
C 1.457805 0.823891 3.147162
C 0.988976 1.167938 4.369361
C -0.456225 1.297942 4.679526
H -3.222205 0.928920 4.756008
C -2.772918 0.691766 3.796110
C -3.559708 0.280010 2.719736
C -2.966488 0.004553 1.488574
N -1.639944 0.106691 1.309297
C -0.864750 0.490772 2.362839
C -1.392280 0.809996 3.615159
Co -0.622492 -0.269595 -0.267525
H -3.535442 -0.307383 0.619676
H -4.634773 0.174846 2.825769
H 1.683052 1.398179 5.172487
H 2.525894 0.756026 2.966923
N 0.840390 0.149444 0.838702
C 2.218216 0.001594 0.409199
O 2.874907 -0.920206 0.851540
C -2.180716 0.840755 -1.827420
O -2.216132 -0.382654 -1.420619
O -1.325850 1.635557 -1.365644
N -0.807863 2.881547 4.879815
O -1.631852 3.132961 5.741967
O -0.257397 3.669968 4.140369
C 2.701357 1.001870 -0.563347
C 3.993961 0.843083 -1.090783
C 4.490055 1.762937 -2.005938
C 3.702029 2.849313 -2.401002
C 2.419043 3.012999 -1.876783
C 1.916053 2.094666 -0.959106
H 4.586780 -0.010182 -0.775953
H 5.487123 1.635305 -2.418025
H 4.088773 3.565647 -3.122099
H 1.801469 3.849785 -2.190597
H 0.907830 2.211574 -0.581428
C 0.434840 -2.145797 -1.406255
O 0.517607 -0.895910 -1.725306
O -0.246616 -2.484340 -0.407369
H -0.719890 0.909849 5.665571
C 1.205317 -3.143253 -2.231267
H 0.766690 -4.138003 -2.128383
H 2.236566 -3.174108 -1.859961
H 1.230781 -2.838675 -3.280824
C -3.171068 1.268468 -2.884014
H -3.381589 2.336737 -2.795475
H -4.092550 0.685448 -2.817213
H -2.725466 1.086197 -3.869406

^{4}Int 2_A

C 0.571732 0.589559 1.989594
C 1.443879 0.958622 3.083847
C 0.956910 1.222477 4.314033
C -0.490896 1.194083 4.633998
H -3.158187 0.479083 4.761743
C -2.717265 0.321680 3.781132
C -3.484603 -0.168328 2.724700
C -2.888900 -0.341664 1.475341
N -1.597884 -0.065162 1.268199
C -0.843022 0.399697 2.286694
C -1.371291 0.622855 3.565297
Co -0.581891 -0.224638 -0.537589
H -3.437477 -0.708119 0.612557
H -4.532141 -0.414745 2.863337
H 1.630649 1.504217 5.118601
H 2.511255 1.005193 2.895507
N 0.927081 0.380354 0.745881
C 2.306241 0.287961 0.365755
O 3.013780 -0.566501 0.871950
C -2.157267 1.079571 -1.950316
O -2.431677 -0.140577 -1.784044
O -1.137863 1.594366 -1.359369
N -1.007637 2.719059 4.865251
O -1.850072 2.862871 5.734025
O -0.551405 3.576368 4.137817
C 2.750364 1.226225 -0.688630
C 4.005434 1.015337 -1.282282
C 4.465671 1.882792 -2.265073
C 3.681357 2.972192 -2.658600
C 2.436610 3.189579 -2.066460
C 1.967559 2.319812 -1.084778
H 4.595977 0.161453 -0.965302
H 5.432278 1.711607 -2.730690
H 4.040523 3.648624 -3.430637
H 1.823517 4.031671 -2.375634
H 0.989248 2.479315 -0.647996
C 0.352012 -2.426697 -1.155042
O 0.769913 -1.315550 -1.640679
O -0.586870 -2.417098 -0.308460
H -0.701892 0.755752 5.611781
C 1.018220 -3.712092 -1.571278
H 0.347082 -4.560119 -1.417763
H 1.910190 -3.853600 -0.950076
H 1.338200 -3.656003 -2.614697
C -2.995940 1.959390 -2.844810
H -3.256352 2.884269 -2.320779
H -3.899086 1.438086 -3.165627
H -2.404950 2.238468 -3.724157

[2]Int 2$_B$
C 0.506921 0.585137 2.029582
C 1.460933 1.038354 3.078826
C 0.856285 1.176009 4.437742
C -0.468438 1.084451 4.661834
H -3.244324 0.840610 4.740610
C -2.795421 0.656840 3.768122
C -3.584584 0.293785 2.674250
C -2.991810 0.042965 1.437765
N -1.664604 0.154335 1.259270
C -0.889738 0.517770 2.319816
C -1.411700 0.770009 3.601717
Co -0.638569 -0.245811 -0.306667
H -3.559966 -0.259909 0.565439
H -4.660786 0.193127 2.774038
H 1.545456 1.397822 5.246818
H 2.387766 0.464017 3.084494
N 0.815204 0.259308 0.788074
C 2.199361 0.162310 0.358114
O 2.906857 -0.680390 0.877960
C -2.219555 0.705173 -1.942505
O -2.228311 -0.484957 -1.446225
O -1.384072 1.549463 -1.536946
N 2.016124 2.529073 2.708239
O 1.310599 3.227654 2.009144
O 3.082643 2.825074 3.210713
C 2.625686 1.097197 -0.697423
C 3.913577 0.939994 -1.238190
C 4.360249 1.802171 -2.230770
C 3.528828 2.830801 -2.687048
C 2.252905 2.995550 -2.146074
C 1.798064 2.133331 -1.153091
H 4.541305 0.133880 -0.871191
H 5.352678 1.675601 -2.654231
H 3.877766 3.503212 -3.467278
H 1.604727 3.790819 -2.502835
H 0.800180 2.255937 -0.752153
C 0.469776 -2.148078 -1.353345
O 0.519677 -0.914325 -1.734155
O -0.203069 -2.455057 -0.338263
H -0.859273 1.219288 5.667696
C 1.263481 -3.162941 -2.133360
H 0.972918 -4.174623 -1.844475
H 2.328764 -3.018130 -1.916978
H 1.116926 -3.014326 -3.207654
C -3.213535 1.031794 -3.030585
H -3.440671 2.100148 -3.027763
H -4.125792 0.441272 -2.918191
H -2.762158 0.778009 -3.997174

[4]Int 2$_B$
C 0.527872 0.659217 1.935087
C 1.432929 1.149362 3.025745
C 0.831699 1.184795 4.390252
C -0.467957 0.924412 4.614844
H -3.156429 0.320439 4.737329
C -2.722820 0.231298 3.744240
C -3.498479 -0.194929 2.665594
C -2.908210 -0.320986 1.407986
N -1.620111 -0.024103 1.204005
C -0.861000 0.399926 2.237622
C -1.371403 0.532187 3.547553
Co -0.574592 -0.221825 -0.571550
H -3.457857 -0.665733 0.536965
H -4.547808 -0.439633 2.794659
H 1.498880 1.461836 5.200783
H 2.393353 0.632193 3.007864
N 0.905253 0.455827 0.700587
C 2.287704 0.436271 0.313931
O 3.051666 -0.331486 0.877660
C -2.182263 0.910082 -2.091004
O -2.377891 -0.319960 -1.909893
O -1.230961 1.507814 -1.459011
N 1.878012 2.670500 2.714186
O 1.153283 3.332241 1.997719
O 2.895145 3.035917 3.271140
C 2.663661 1.316024 -0.810617
C 3.914071 1.118729 -1.419696
C 4.310883 1.933663 -2.471943
C 3.468980 2.958342 -2.917303
C 2.231308 3.165118 -2.306692
C 1.824258 2.346244 -1.256702
H 4.550471 0.316417 -1.059691
H 5.272926 1.772954 -2.950603
H 3.779324 3.594321 -3.742916
H 1.576557 3.960639 -2.651520
H 0.857481 2.503136 -0.795687
C 0.376410 -2.444423 -1.088555
O 0.769781 -1.360027 -1.645844
O -0.536900 -2.395419 -0.214012
H -0.866767 0.976217 5.625458
C 1.034404 -3.747528 -1.460473
H 0.419788 -4.593511 -1.146271
H 2.004565 -3.804440 -0.953931
H 1.217629 -3.782874 -2.537937
C -3.026180 1.715603 -3.047725
H -3.326147 2.659719 -2.584000
H -3.903341 1.148337 -3.363306
H -2.421894 1.960838 -3.928633

[5]Int 3$_A$
C -1.88403 0.792797 0.125137
C -1.29167 1.952612 0.731663
C -0.26595 1.825987 1.605449
C 0.282875 0.511415 2.038951
H 1.100707 -2.12542 2.134293
C 0.239203 -1.96762 1.494344
C -0.34845 -3.01445 0.787772
C -1.43652 -2.77166 -0.0476
N -1.93273 -1.53598 -0.21533

C -1.35009 -0.50389 0.459157
C -0.2818 -0.68133 1.339693
Co -3.32225 -0.98193 -1.40622
H -1.92534 -3.55448 -0.61672
H 0.044411 -4.02296 0.866916
H 0.204059 2.703475 2.036607
H -1.64085 2.935589 0.435826
N -2.88033 0.749525 -0.75174
C -3.45953 1.952264 -1.27143
O -2.78329 2.738535 -1.91183
C -4.8374 -2.90038 -0.9418
O -3.88896 -2.82355 -1.80953
O -5.06604 -1.96507 -0.14058
N 0.018771 0.416976 3.619815
O -0.73581 -0.44164 4.036214
O 0.595065 1.255797 4.294127
C -4.9009 2.160592 -0.97607
C -5.5325 3.288877 -1.52272
C -6.8704 3.545698 -1.24555
C -7.59143 2.678217 -0.41852
C -6.96783 1.554562 0.125929
C -5.62675 1.294633 -0.14694
H -4.95359 3.949074 -2.16136
H -7.35457 4.420363 -1.67307
H -8.63869 2.877826 -0.20308
H -7.52607 0.869189 0.758183
H -5.15931 0.40515 0.257602
C -3.62281 -0.27215 -3.73785
O -4.51126 -0.28474 -2.80095
O -2.45315 -0.65711 -3.51124
H 1.375736 0.500724 2.008518
C 6.144367 -1.06709 -0.33186
O 5.499379 -1.90798 0.386321
O 5.717149 0.105973 -0.49791
C 7.42992 -1.52679 -0.99512
H 8.080218 -2.00407 -0.25398
H 7.946874 -0.68625 -1.46411
H 7.190655 -2.27878 -1.7562
C 2.171413 -0.19257 -0.79195
O 2.604827 -1.36947 -0.64086
O 2.632751 0.758694 -0.06858
C 1.134432 0.115011 -1.84987
H 0.570596 1.017794 -1.60509
H 0.45485 -0.72777 -1.9941
H 1.653477 0.289228 -2.80023
Co 4.031583 -0.52262 0.8431
C 3.872884 -0.3169 3.345809
O 2.975591 -0.99729 2.742941
O 4.779109 0.288831 2.700884
C 3.824479 -0.2193 4.856785
H 4.781138 0.125326 5.255471
H 3.555796 -1.18786 5.288653
H 3.042817 0.496692 5.135953
C -4.03174 0.268822 -5.0896
H -3.49081 -0.25416 -5.8818
H -3.75799 1.329948 -5.13111
H -5.11157 0.181772 -5.23595
C -5.69022 -4.15344 -0.94368
H -6.01118 -4.39089 0.073524
H -5.15295 -4.99721 -1.3843
H -6.58527 -3.96214 -1.54767

^{5}Int 3$_{B}$

C 0.994532 0.134611 0.501308
C 2.185092 0.702273 1.20537
C 2.396793 0.177173 2.588622
C 1.580082 -0.71653 3.169996
H -0.17456 -2.69011 3.965945
C -0.39905 -2.26651 2.993297
C -1.44783 -2.74525 2.208297
C -1.6692 -2.22519 0.933733
N -0.87791 -1.27064 0.419031
C 0.163628 -0.81135 1.170986
C 0.429714 -1.26474 2.476156
Co -0.95978 -0.48966 -1.32415
H -2.47056 -2.57087 0.289929
H -2.09362 -3.53927 2.571472
H 3.297881 0.507477 3.093581
H 3.089216 0.614108 0.599448
N 0.60298 0.429195 -0.72557
C 1.382044 1.283917 -1.58025
O 2.512812 0.955751 -1.89426
C -3.43739 -0.28014 -1.36838
O -2.7129 -1.28161 -1.72956
O -2.92653 0.745938 -0.86462
N 2.029028 2.288209 1.361578
O 0.901343 2.740863 1.461989
O 3.069624 2.921354 1.429491
C 0.727119 2.532303 -2.0374
C 1.41271 3.335916 -2.96159
C 0.852742 4.530272 -3.3993
C -0.39509 4.934921 -2.91444
C -1.07755 4.140623 -1.99197
C -0.52132 2.943081 -1.55112
H 2.380464 3.003516 -3.32226
H 1.386011 5.149277 -4.11706
H -0.8325 5.869681 -3.25724
H -2.04928 4.447508 -1.61623
H -1.06376 2.323203 -0.84819
C -0.19337 -0.83662 -3.64097
O -0.82139 0.183066 -3.16212
O 0.094305 -1.80952 -2.90703
H 1.820448 -1.11803 4.147707
C 5.578639 -2.29592 3.530718
O 4.513113 -1.83032 4.038997
O 5.579421 -3.39439 2.891002
C 2.34959 -3.47231 0.90658
O 3.112378 -2.49698 1.143538
O 2.171234 -4.38435 1.794252
Co 3.475616 -3.5591 3.149508

C 6.872262 -1.51325 3.654627
H 7.731385 -2.18922 3.668128
H 6.861983 -0.88605 4.550358
H 6.966633 -0.85953 2.778352
C 1.637464 -3.5997 -0.4196
H 2.141393 -4.37019 -1.01564
H 1.652944 -2.66267 -0.97572
H 0.604723 -3.92543 -0.26992
C 2.522009 -4.45536 5.289259
O 3.552943 -4.95269 4.74046
O 1.901838 -3.48289 4.764125
C 2.029951 -5.02056 6.611214
H 2.172857 -4.26816 7.396174
H 2.575478 -5.92856 6.877517
H 0.957279 -5.23217 6.548538
C -4.93196 -0.3805 -1.60136
H -5.47035 0.22275 -0.86657
H -5.26528 -1.42105 -1.56487
H -5.15566 0.013319 -2.60019
C 0.222841 -0.78664 -5.09435
H 0.28867 -1.79767 -5.50338
H 1.21675 -0.32626 -5.14971
H -0.47064 -0.17637 -5.67832

^{5}TS 3-4$_{A}$
C -1.72115 -0.17895 -0.38369
C -0.72667 0.849955 -0.36858
C 0.273764 0.829706 0.54745
C 0.467142 -0.27427 1.499634
H 0.194022 -2.53822 3.132943
C -0.60329 -2.41071 2.41174
C -1.6248 -3.34866 2.311092
C -2.64143 -3.18367 1.371738
N -2.65072 -2.13938 0.531832
C -1.64179 -1.22165 0.61465
C -0.61392 -1.2934 1.562158
Co -3.92535 -1.7643 -0.83992
H -3.46226 -3.88378 1.263398
H -1.63896 -4.22075 2.958336
H 1.013699 1.61981 0.559747
H -0.78026 1.652726 -1.09679
N -2.76396 -0.2933 -1.18968
C -2.95887 0.615732 -2.28121
O -2.23287 0.57401 -3.25655
C -5.91947 -2.35911 0.541102
O -5.20274 -3.12147 -0.21023
O -5.63134 -1.15459 0.721917
N 0.954344 0.228127 2.830334
O 0.481617 -0.2159 3.871122
O 1.86976 1.076636 2.816539
C -4.08479 1.576543 -2.13304
C -4.31533 2.491708 -3.17223
C -5.33736 3.428249 -3.06708
C -6.14054 3.458904 -1.92236
C -5.9164 2.549339 -0.88771
C -4.89139 1.610834 -0.98755
H -3.68037 2.448719 -4.05222
H -5.5118 4.135273 -3.87503
H -6.94216 4.189989 -1.84044
H -6.54527 2.561259 -0.00121
H -4.7445 0.887281 -0.19449
C -4.40584 -2.23214 -3.20518
O -5.02504 -1.41189 -2.4229
O -3.44832 -2.91808 -2.78287
H 1.451836 -0.93775 1.099861
C 4.343595 0.880281 -0.76256
O 5.337859 0.73097 -0.0163
O 3.174187 1.112868 -0.27773
C 4.461185 0.787499 -2.27181
H 4.034432 1.684782 -2.73269
H 3.875998 -0.06859 -2.6269
H 5.504091 0.671714 -2.57422
C 4.583332 3.163409 2.675662
O 3.711654 3.334806 1.784934
O 4.974827 1.985183 2.991446
C 5.203325 4.349832 3.389943
H 6.058256 4.706858 2.802703
H 5.563743 4.060925 4.380162
H 4.478903 5.165168 3.46767
Co 3.721435 1.051171 1.676916
C 3.468154 -1.91563 1.356451
O 2.368322 -1.96159 0.719491
O 3.885962 -0.94664 2.049316
C 4.374701 -3.13408 1.268133
H 4.737131 -3.39649 2.266363
H 5.247702 -2.86192 0.664053
H 3.863332 -3.98338 0.810183
C -4.84663 -2.28975 -4.65138
H -4.64721 -3.28114 -5.06657
H -4.2614 -1.55499 -5.21671
H -5.90587 -2.03707 -4.749
C -7.15042 -2.97508 1.179412
H -7.40437 -2.44487 2.10036
H -6.99832 -4.03964 1.377543
H -7.99008 -2.87653 0.480349

^{4}TS 3-4$_{B}$
C 0.235041 -0.21306 1.60291
C 1.597583 -0.31186 2.185253
C 1.775381 -1.35058 3.226548
C 0.822149 -2.24631 3.552291
H -1.3801 -3.87414 3.980075
C -1.53897 -3.07866 3.25606
C -2.76683 -2.93024 2.612662
C -2.94736 -1.906 1.686243
N -1.96122 -1.04048 1.390654
C -0.75055 -1.18116 2.00692
C -0.49428 -2.19249 2.95501
Co -1.99449 0.321309 0.073425
H -3.87809 -1.75536 1.151457

H -3.5917 -3.60565 2.821157
H 2.762354 -1.3861 3.675964
H 2.390409 -0.66509 1.325424
N -0.20442 0.630912 0.689888
C 0.668299 1.562773 0.006355
O 1.563353 1.117976 -0.68325
C -4.35733 1.047875 0.433821
O -3.91305 0.100041 -0.31747
O -3.59766 1.70323 1.180723
N 2.186479 0.992566 2.681367
O 1.489776 1.995368 2.713478
O 3.378384 0.946531 3.026746
C 0.364115 3.003918 0.148813
C 1.259348 3.913885 -0.43195
C 1.032879 5.281108 -0.32114
C -0.09129 5.749169 0.364436
C -0.98059 4.844455 0.949732
C -0.7518 3.47545 0.851986
H 2.13921 3.527532 -0.93268
H 1.738738 5.983091 -0.75802
H -0.26933 6.819649 0.450325
H -1.8564 5.20478 1.483854
H -1.44846 2.779448 1.303018
C -1.44447 0.607553 -2.29863
O -1.80922 1.520328 -1.46343
O -1.37248 -0.59098 -1.93903
H 1.028911 -3.02895 4.278885
C 6.65508 0.793901 3.567271
O 6.239933 1.985107 3.503881
O 6.302971 -0.07126 2.697768
C 5.202068 3.52181 0.455897
O 6.274457 2.855419 0.556491
O 4.127312 3.11331 0.998948
Co 5.152261 1.381658 1.683217
C 7.610804 0.38547 4.674199
H 7.361935 0.908168 5.602216
H 8.628168 0.679301 4.387404
H 7.592277 -0.69689 4.826636
C 5.176373 4.813114 -0.34105
H 4.601502 5.572468 0.197889
H 4.669815 4.629057 -1.29621
H 6.189203 5.16965 -0.54085
C 4.122295 -0.76407 -0.2626
O 4.678776 0.326299 0.044588
O 3.241352 -1.38604 0.401711
C 4.515233 -1.3767 -1.60137
H 4.442391 -2.4673 -1.56348
H 5.520403 -1.06206 -1.89076
H 3.802408 -1.01345 -2.35183
C -5.83985 1.3609 0.356819
H -6.20005 1.718511 1.324918
H -6.41268 0.488142 0.031911
H -5.9864 2.161601 -0.37837
C -1.04946 1.046015 -3.69018
H -1.227 0.240115 -4.40721
H 0.023618 1.272295 -3.67846
H -1.58885 1.950584 -3.98267

^{4}Int 4$_A$

C 0.544543 0.751768 2.261531
C 1.307334 1.172907 3.371508
C 0.723691 1.337434 4.614629
C -0.631080 1.084968 4.858506
H -3.367593 0.439500 4.801258
C -2.858496 0.334957 3.854803
C -3.535384 -0.087573 2.725353
C -2.856655 -0.212519 1.509472
N -1.560648 0.064540 1.407625
C -0.859749 0.480131 2.497210
C -1.474036 0.640077 3.782830
Co -0.454773 -0.064789 -0.311811
H -3.346383 -0.533167 0.593979
H -4.595436 -0.321741 2.776639
H 1.325991 1.672165 5.450940
H 2.364566 1.363859 3.244424
N 0.983418 0.639655 0.984331
C 2.342963 0.601724 0.687053
O 3.203478 0.120863 1.427511
C -2.154692 1.138810 -1.769304
O -2.195974 -0.124540 -1.689470
O -1.317498 1.820557 -1.101206
N -1.096588 1.293139 6.209728
O -2.293661 1.090578 6.488263
O -0.280839 1.676613 7.066874
C 2.724685 1.176370 -0.645208
C 3.948134 0.797284 -1.213024
C 4.356783 1.331152 -2.431281
C 3.553296 2.266572 -3.089404
C 2.341891 2.660850 -2.519922
C 1.926972 2.118669 -1.305049
H 4.560293 0.073564 -0.683176
H 5.300019 1.016427 -2.872252
H 3.868827 2.681018 -4.044397
H 1.707086 3.383329 -3.027908
H 0.973650 2.411512 -0.881892
C 0.660408 -1.997059 -1.429380
O 0.824348 -0.895699 -2.011898
O -0.018909 -2.088791 -0.343852
C 1.291463 -3.264329 -1.978693
H 0.562002 -4.080998 -1.973704
H 2.121729 -3.557295 -1.325167
H 1.671128 -3.099738 -2.989517
C -3.109416 1.858502 -2.708577
H -3.343039 2.857528 -2.330870
H -4.024853 1.277446 -2.848782
H -2.621625 1.969587 -3.684788

^{4}Int 4$_B$

C 0.508577 0.888558 2.460533
C 1.094134 1.308457 3.686574

C 0.378107 1.263891 4.903792
C -0.943821 0.899945 4.960468
H -3.575318 0.194263 4.632487
C -2.995221 0.199935 3.711983
C -3.574970 -0.130462 2.500599
C -2.791208 -0.108904 1.337677
N -1.498975 0.215536 1.360410
C -0.903255 0.549451 2.536811
C -1.626042 0.552933 3.763240
Co -0.230320 0.195183 -0.232532
H -3.196421 -0.353358 0.359921
H -4.624326 -0.405410 2.435120
H 0.912256 1.562016 5.798739
H -1.479535 0.893732 5.906326
N 1.075374 0.773979 1.243290
C 2.437721 0.519692 1.076393
O 3.076211 -0.219335 1.821546
C -1.720909 1.549042 -1.779934
O -1.882814 0.295129 -1.774512
O -0.891604 2.119096 -1.002980
N 2.418149 1.907751 3.765876
O 3.027984 1.835764 4.842358
O 2.848109 2.518070 2.783620
C 3.063109 1.144366 -0.131668
C 4.293683 0.652599 -0.584168
C 4.918766 1.226031 -1.687874
C 4.324330 2.307770 -2.343722
C 3.104711 2.810490 -1.886789
C 2.475588 2.232643 -0.786362
H 4.740021 -0.184390 -0.055604
H 5.868794 0.830601 -2.040423
H 4.809646 2.754671 -3.209161
H 2.635294 3.650059 -2.394322
H 1.522637 2.614528 -0.440910
C 0.856825 -1.739177 -1.374879
O 1.142315 -0.620322 -1.868694
O 0.069548 -1.849114 -0.366367
C -2.506816 2.403672 -2.762704
H -2.664168 3.409959 -2.364455
H -3.463626 1.933451 -3.005470
H -1.924833 2.490105 -3.688853
C 1.447823 -3.014210 -1.950148
H 0.639516 -3.666123 -2.300937
H 1.985158 -3.552377 -1.161567
H 2.126978 -2.787683 -2.775143

Napthylpicolinamide Structures

^{1}Int 1
Co -1.48601 -4.23354 -3.94232
N -0.59299 -4.90831 -2.39456
C 0.718225 -4.6011 -2.27188
O 1.483761 -4.91194 -1.35711
C -3.26546 -3.33255 -2.75381
O -2.18788 -2.67947 -2.99763
O -3.31671 -4.51404 -3.22666
C -1.96951 -5.19717 -6.03227
O -1.33547 -5.80632 -5.10099
O -2.27984 -3.98123 -5.82254
C 1.175827 -3.79854 -3.45578
C 2.47219 -3.31772 -3.59711
C 2.793297 -2.56883 -4.7266
C 1.802189 -2.31914 -5.67781
C 0.523453 -2.82863 -5.47802
N 0.230384 -3.55634 -4.38723
H 3.186478 -3.54587 -2.81227
H 3.798182 -2.17984 -4.86414
H 2.008787 -1.73568 -6.56941
H -0.28848 -2.66512 -6.17801
C -2.35227 -5.9121 -7.29376
H -2.39809 -5.20867 -8.12873
H -3.34875 -6.34863 -7.15849
H -1.64768 -6.71979 -7.50414
C -4.36156 -2.75592 -1.91905
H -5.32382 -3.18395 -2.20902
H -4.37748 -1.66675 -2.00745
H -4.16188 -3.01639 -0.87234
C -1.28835 -5.65327 -1.42676
C -1.61493 -6.97089 -1.69893
C -2.38227 -7.72719 -0.79243
C -2.83335 -7.1609 0.380404
H -3.43442 -7.73913 1.078925
H -3.59146 -5.79243 2.579773
C -2.99172 -5.20084 1.891187
C -2.69048 -3.88741 2.178257
C -1.90099 -3.13019 1.283198
C -1.43115 -3.69359 0.115759
C -1.73168 -5.03812 -0.21303
C -2.52661 -5.81274 0.697762
H -0.83122 -3.10859 -0.57165
H -1.66133 -2.09663 1.518623
H -3.05405 -3.43269 3.096056
H -2.62198 -8.76063 -1.0277
H -1.28017 -7.4023 -2.6367

^{3}Int 1
Co -1.41131 -4.13123 -3.77853
N -0.51618 -4.86281 -2.31861
C 0.855744 -4.74071 -2.30704
O 1.619949 -5.19037 -1.46352
C -3.39701 -3.23448 -2.72624
O -2.48583 -2.39228 -2.97985
O -3.21412 -4.46192 -3.05584
C -1.99892 -5.10808 -5.98198
O -1.40769 -5.90079 -5.20549
O -2.1842 -3.88608 -5.62409
C 1.32501 -3.94245 -3.48774
C 2.654419 -3.61375 -3.71373
C 2.971643 -2.85734 -4.84127
C 1.94604 -2.45599 -5.69817

C 0.633951 -2.82362 -5.4129
N 0.343139 -3.55552 -4.32627
H 3.399396 -3.9564 -3.00291
H 4.001663 -2.58198 -5.04862
H 2.15176 -1.86378 -6.58415
H -0.20572 -2.54848 -6.04174
C -2.52878 -5.56207 -7.31939
H -2.5641 -4.73083 -8.02824
H -3.55059 -5.93281 -7.17731
H -1.91868 -6.38014 -7.70942
C -4.66108 -2.85031 -2.01002
H -5.49026 -3.48695 -2.32727
H -4.89102 -1.79683 -2.18363
H -4.50184 -3.00159 -0.93532
C -1.17085 -5.63798 -1.33732
C -1.32098 -6.99864 -1.53384
C -2.03358 -7.77969 -0.60362
C -2.59806 -7.1964 0.510633
H -3.15715 -7.79712 1.224528
H -3.59922 -5.78659 2.587463
C -3.04539 -5.17507 1.87828
C -2.9072 -3.81937 2.084187
C -2.17771 -3.03333 1.163861
C -1.60181 -3.61139 0.05202
C -1.73379 -5.00012 -0.19036
C -2.46767 -5.80371 0.744937
H -1.05532 -3.00255 -0.65987
H -2.07226 -1.96457 1.332305
H -3.35569 -3.35157 2.95684
H -2.14279 -8.84704 -0.77716
H -0.90318 -7.44639 -2.42995

^{5}Int 1

Co -1.57496 -4.41403 -3.78262
N -0.4414 -5.03883 -2.34975
C 0.862353 -4.65563 -2.22371
O 1.619743 -4.92412 -1.29631
C -3.52703 -3.28902 -2.8251
O -2.4458 -2.64828 -3.04986
O -3.60464 -4.50918 -3.17931
C -2.11249 -5.22158 -6.06613
O -1.76059 -6.00391 -5.12397
O -2.13561 -3.96764 -5.85987
C 1.299245 -3.82385 -3.40415
C 2.597395 -3.33704 -3.52296
C 2.916502 -2.56266 -4.63762
C 1.928473 -2.30066 -5.58621
C 0.650438 -2.82533 -5.39435
N 0.353846 -3.57233 -4.32561
H 3.316868 -3.57288 -2.74571
H 3.92024 -2.1661 -4.76324
H 2.13674 -1.69816 -6.46509
H -0.16184 -2.65836 -6.09514
C -2.53108 -5.78868 -7.39846
H -2.36341 -5.06068 -8.19582
H -3.6034 -6.01348 -7.35674
H -1.99675 -6.71902 -7.60432
C -4.67057 -2.62425 -2.10889
H -5.62385 -3.04486 -2.43674
H -4.6478 -1.54324 -2.26626
H -4.55928 -2.82101 -1.03494
C -1.14472 -5.74948 -1.37291
C -1.47563 -7.07934 -1.61901
C -2.23919 -7.81026 -0.69541
C -2.69249 -7.21256 0.464502
H -3.29065 -7.77587 1.177079
H -3.47685 -5.7882 2.617075
C -2.88925 -5.20863 1.908569
C -2.62757 -3.8744 2.142868
C -1.86836 -3.12915 1.216223
C -1.37417 -3.72715 0.073406
C -1.61977 -5.09368 -0.18907
C -2.40464 -5.85099 0.741464
H -0.80943 -3.14408 -0.64508
H -1.67379 -2.07616 1.400878
H -3.00891 -3.3939 3.039944
H -2.47641 -8.84973 -0.90313
H -1.13678 -7.53273 -2.54489

^{2}TS 1-2$_A$

Co -1.678121 -4.092205 -4.160696
N -0.837557 -4.766895 -2.552456
C 0.445708 -4.357122 -2.351466
O 1.161769 -4.586627 -1.378417
C -3.450666 -3.125825 -3.000510
O -2.344798 -2.512577 -3.215422
O -3.540898 -4.300855 -3.484385
C -2.145009 -5.057878 -6.266209
O -1.553678 -5.671054 -5.307453
O -2.425716 -3.831762 -6.089765
C 0.948954 -3.605419 -3.548675
C 2.241216 -3.100044 -3.634179
C 2.623824 -2.419718 -4.787583
C 1.695646 -2.261043 -5.818172
C 0.416352 -2.786237 -5.669172
N 0.062330 -3.447463 -4.554171
H 2.906434 -3.258667 -2.791395
H 3.627544 -2.015803 -4.883107
H 1.950727 -1.735028 -6.732553
H -0.350379 -2.684838 -6.429242
C -2.506398 -5.789887 -7.525227
H -2.516880 -5.101657 -8.373932
H -3.513842 -6.206622 -7.410118
H -1.811296 -6.613665 -7.701542
C -4.538966 -2.513183 -2.179657
H -5.508585 -2.927376 -2.464777
H -4.533761 -1.425630 -2.288433
H -4.348921 -2.755607 -1.126437
C -1.581381 -5.446111 -1.609280
C -2.110954 -6.706259 -1.964386

C -2.871806 -7.442193 -1.087211
C -3.090257 -6.983632 0.247376
H -3.915693 -7.412742 0.810095
H -3.786108 -5.590499 2.444406
C -3.181317 -5.006286 1.756083
C -2.833377 -3.698696 2.064127
C -2.037368 -2.957737 1.175287
C -1.600364 -3.524156 -0.009864
C -1.942540 -4.852295 -0.345846
C -2.748046 -5.602604 0.560824
H -1.005597 -2.941893 -0.702792
H -1.765718 -1.933543 1.415056
H -3.176875 -3.249962 2.992017
H -3.247865 -8.417816 -1.378925
H -1.896338 -7.076266 -2.961600
N -1.780517 -7.979499 1.328572
O -2.068697 -8.053503 2.504501
O -0.798819 -8.397678 0.765961

^{4}TS 1-2$_A$

Co -1.75791 -4.25024 -4.00676
N -0.66979 -4.96951 -2.53137
C 0.604003 -4.47757 -2.35947
O 1.338178 -4.7024 -1.40668
C -3.67644 -3.13188 -2.93153
O -2.62126 -2.48013 -3.24165
O -3.76419 -4.36245 -3.22756
C -2.28417 -5.1043 -6.27093
O -1.93922 -5.89973 -5.34083
O -2.38478 -3.85959 -6.02266
C 1.042743 -3.65954 -3.54367
C 2.328957 -3.14062 -3.64735
C 2.649828 -2.38268 -4.77303
C 1.671778 -2.16306 -5.74298
C 0.402571 -2.71088 -5.5628
N 0.10762 -3.44968 -4.48604
H 3.039311 -3.33945 -2.85156
H 3.646014 -1.96553 -4.89005
H 1.881316 -1.57472 -6.631
H -0.40429 -2.57265 -6.27616
C -2.51459 -5.61871 -7.67053
H -1.53421 -5.74892 -8.14687
H -3.0934 -4.90309 -8.25595
H -2.99894 -6.59885 -7.65253
C -4.80221 -2.46927 -2.17602
H -5.75585 -2.69117 -2.6636
H -4.65343 -1.38896 -2.11256
H -4.83847 -2.88861 -1.16453
C -1.44413 -5.57161 -1.59863
C -1.94936 -6.87295 -1.8958
C -2.7 -7.56451 -0.98963
C -2.99776 -7.01005 0.296794
H -3.84525 -7.42435 0.8388
H -3.74369 -5.50926 2.411323
C -3.12773 -4.96412 1.702327
C -2.78907 -3.63776 1.944313
C -1.99382 -2.93616 1.028513
C -1.53869 -3.56196 -0.12217
C -1.85572 -4.90902 -0.37857
C -2.67198 -5.618 0.54824
H -0.9608 -3.00388 -0.8489
H -1.74262 -1.89544 1.211012
H -3.14885 -3.14339 2.842423
H -3.03599 -8.57062 -1.21758
H -1.69947 -7.29387 -2.86394
N -1.70462 -8.00025 1.535679
O -1.71098 -7.60776 2.677327
O -1.04872 -8.87693 1.034115

^{2}TS 1-2$_B$

Co -1.479777 -4.353463 -4.168910
N -0.482911 -5.117487 -2.636670
C 0.761874 -4.565254 -2.483268
O 1.551264 -4.766231 -1.560801
C -3.338559 -3.769342 -2.896401
O -2.305624 -3.015953 -2.990888
O -3.291421 -4.866211 -3.542435
C -1.913710 -5.064778 -6.391189
O -1.203002 -5.724515 -5.553658
O -2.335821 -3.922386 -6.032292
C 1.124560 -3.678775 -3.631739
C 2.368581 -3.065322 -3.738860
C 2.615879 -2.228702 -4.823369
C 1.602091 -2.022661 -5.760680
C 0.378220 -2.662005 -5.593239
N 0.156346 -3.478977 -4.548992
H 3.101620 -3.261142 -2.963097
H 3.578929 -1.738997 -4.935208
H 1.748460 -1.372802 -6.617749
H -0.447693 -2.532457 -6.283576
C -2.250216 -5.655747 -7.729368
H -2.372259 -4.865287 -8.473595
H -3.201717 -6.193639 -7.641942
H -1.480009 -6.366122 -8.039424
C -4.505846 -3.406738 -2.035785
H -5.431421 -3.781637 -2.479426
H -4.554534 -2.325627 -1.889470
H -4.375257 -3.891368 -1.060641
C -1.142984 -5.713827 -1.591442
C -1.117547 -5.129263 -0.274692
C -2.194314 -5.463508 0.635659
C -3.019515 -6.511021 0.379497
H -3.799260 -6.784944 1.086715
H -4.398241 -8.725648 -0.296236
C -3.675942 -8.427556 -1.053336
C -3.539030 -9.166372 -2.220136
C -2.598971 -8.778818 -3.190946
C -1.814432 -7.656348 -2.995167
C -1.936109 -6.887094 -1.816634
C -2.883246 -7.289425 -0.821990

H -1.094211 -7.354744 -3.745937
H -2.483655 -9.366144 -4.098213
H -4.155167 -10.047335 -2.380068
H -2.277550 -4.894447 1.557251
H -0.678560 -4.140575 -0.194846
N 0.375677 -5.967761 0.686495
O 0.864398 -5.247663 1.523872
O 0.592551 -7.127562 0.450118

^{4}TS 1-2$_B$
Co -1.54924 -4.45194 -4.08652
N -0.37443 -5.16562 -2.61329
C 0.849286 -4.56865 -2.43958
O 1.602689 -4.71833 -1.48106
C -3.60762 -3.71798 -2.9503
O -2.59479 -2.94519 -3.05265
O -3.5729 -4.86704 -3.48844
C -2.06565 -5.05899 -6.43451
O -1.59576 -5.89744 -5.59702
O -2.21072 -3.8461 -6.08812
C 1.236006 -3.69374 -3.60152
C 2.504418 -3.12867 -3.69929
C 2.779865 -2.29803 -4.78392
C 1.776247 -2.05368 -5.72097
C 0.529168 -2.65288 -5.54942
N 0.275845 -3.45993 -4.51198
H 3.234865 -3.3463 -2.92729
H 3.760246 -1.84285 -4.89354
H 1.948587 -1.40623 -6.57531
H -0.2943 -2.49974 -6.24005
C -2.47482 -5.52387 -7.8093
H -2.39959 -4.70677 -8.53106
H -3.5215 -5.84758 -7.7662
H -1.86711 -6.3759 -8.12318
C -4.81243 -3.29697 -2.14903
H -5.71269 -3.78258 -2.53136
H -4.92434 -2.21004 -2.15463
H -4.66221 -3.6233 -1.11231
C -1.10583 -5.70168 -1.58911
C -1.2289 -5.02341 -0.34511
C -2.2423 -5.39233 0.575599
C -3.01279 -6.50094 0.347243
H -3.78189 -6.79417 1.057833
H -4.30237 -8.77656 -0.28406
C -3.56445 -8.48471 -1.02802
C -3.36096 -9.26172 -2.1563
C -2.40067 -8.88211 -3.11061
C -1.65991 -7.72701 -2.93504
C -1.85228 -6.91595 -1.79713
C -2.81815 -7.30661 -0.81823
H -0.93467 -7.42691 -3.68156
H -2.24186 -9.49432 -3.99392
H -3.94414 -10.1668 -2.30347
H -2.37608 -4.79501 1.472781
H -0.70209 -4.0853 -0.22221
N 0.557335 -6.07424 0.754205
O 1.045833 -5.33246 1.561368
O 0.710982 -7.24505 0.544892

^{2}Int 2$_A$
Co -1.49169 -4.27391 -4.00624
N -0.63772 -4.95204 -2.37955
C 0.633017 -4.48926 -2.17483
O 1.350359 -4.69193 -1.19818
C -3.27479 -3.31697 -2.84407
O -2.16861 -2.70055 -3.04575
O -3.36382 -4.48828 -3.33655
C -1.95515 -5.24425 -6.12559
O -1.36099 -5.84756 -5.16062
O -2.24419 -4.01974 -5.9687
C 1.122768 -3.739 -3.37724
C 2.403876 -3.20413 -3.45676
C 2.782511 -2.52944 -4.61467
C 1.861063 -2.40593 -5.65602
C 0.592521 -2.95792 -5.51222
N 0.241167 -3.61333 -4.39255
H 3.064868 -3.33622 -2.60621
H 3.777562 -2.10366 -4.70534
H 2.113066 -1.88656 -6.57505
H -0.16943 -2.88316 -6.28016
C -2.31454 -5.9969 -7.37473
H -2.34194 -5.31872 -8.23101
H -3.31462 -6.42744 -7.24668
H -1.60932 -6.81287 -7.54793
C -4.36877 -2.70919 -2.02529
H -5.33532 -3.13144 -2.30895
H -4.37194 -1.62207 -2.13904
H -4.17817 -2.94393 -0.97034
C -1.38423 -5.6003 -1.4396
C -1.9843 -6.84015 -1.81264
C -2.71013 -7.58362 -0.93917
C -2.87848 -7.16928 0.460817
H -3.84213 -7.44362 0.892784
H -3.52435 -5.74364 2.651449
C -2.92336 -5.16396 1.955823
C -2.56832 -3.85323 2.26299
C -1.78759 -3.11654 1.36398
C -1.37068 -3.6871 0.170895
C -1.7148 -5.01511 -0.15595
C -2.50596 -5.75558 0.760694
H -0.79454 -3.1025 -0.53525
H -1.51197 -2.09146 1.595043
H -2.90072 -3.40604 3.195471
H -3.12555 -8.54064 -1.24009
H -1.82537 -7.17588 -2.83255
N -1.885 -8.11284 1.413474
O -2.34718 -8.42956 2.496404
O -0.78475 -8.37975 0.98165

^{4}Int 2$_A$

Co -1.43942 -4.50776 -3.86721
N -0.30742 -5.2411 -2.37672
C 0.911303 -4.62958 -2.15731
O 1.638561 -4.81851 -1.19329
C -3.40276 -3.45934 -2.73268
O -2.32143 -2.83995 -3.04935
O -3.55046 -4.67052 -3.04589
C -2.10713 -5.37632 -6.08232
O -1.72515 -6.1288 -5.12298
O -2.08806 -4.11802 -5.9305
C 1.312272 -3.75723 -3.31578
C 2.550416 -3.12522 -3.36583
C 2.841932 -2.32164 -4.46745
C 1.883526 -2.17351 -5.47002
C 0.66127 -2.83218 -5.3427
N 0.39265 -3.60978 -4.28684
H 3.248651 -3.27538 -2.5489
H 3.800578 -1.81594 -4.54155
H 2.072166 -1.55531 -6.34233
H -0.12966 -2.75409 -6.08277
C -2.60824 -5.99595 -7.36241
H -2.57871 -5.27164 -8.1794
H -3.64748 -6.31071 -7.21169
H -2.02316 -6.88486 -7.61232
C -4.45789 -2.72247 -1.94382
H -5.42837 -3.21255 -2.04703
H -4.51712 -1.67912 -2.26403
H -4.16968 -2.73417 -0.8853
C -1.15726 -5.76116 -1.48722
C -1.77584 -7.01944 -1.82883
C -2.59275 -7.66648 -0.97639
C -2.89138 -7.13297 0.367735
H -3.92457 -7.30665 0.672075
H -3.63467 -5.55001 2.409544
C -2.95375 -5.04069 1.7325
C -2.56823 -3.7268 1.984671
C -1.69739 -3.0756 1.105127
C -1.21561 -3.73742 -0.01478
C -1.57976 -5.07095 -0.27448
C -2.47105 -5.71887 0.611624
H -0.58176 -3.20913 -0.71506
H -1.40772 -2.04502 1.287145
H -2.95376 -3.20785 2.857717
H -3.02012 -8.62891 -1.24198
H -1.54735 -7.42257 -2.80984
N -2.09493 -8.04407 1.461835
O -2.75868 -8.4416 2.403762
O -0.91224 -8.22935 1.269188

^{2}Int 2$_{B}$

Co -1.45806 -4.40715 -4.19088
N -0.42418 -5.17339 -2.63044
C 0.803171 -4.55118 -2.48346
O 1.608191 -4.74926 -1.57678
C -3.34702 -3.8727 -2.93005
O -2.3239 -3.10291 -2.98214
O -3.27299 -4.95355 -3.59982
C -1.86418 -5.12815 -6.44098
O -1.14772 -5.76034 -5.58349
O -2.32197 -3.99221 -6.12146
C 1.111749 -3.62274 -3.6091
C 2.314621 -2.92654 -3.68535
C 2.527295 -2.06322 -4.75588
C 1.52087 -1.91658 -5.71209
C 0.337917 -2.63537 -5.57423
N 0.146654 -3.47489 -4.54134
H 3.046467 -3.08213 -2.89953
H 3.457889 -1.50979 -4.84273
H 1.641514 -1.2519 -6.56186
H -0.48184 -2.55471 -6.27927
C -2.1675 -5.76345 -7.76962
H -2.38386 -4.99692 -8.51675
H -3.05496 -6.39748 -7.6562
H -1.33661 -6.39631 -8.0924
C -4.5392 -3.54505 -2.0878
H -5.44024 -3.98401 -2.52235
H -4.64651 -2.46336 -1.97937
H -4.3888 -3.98354 -1.09371
C -1.03411 -5.77601 -1.59413
C -0.87745 -5.2445 -0.20716
C -2.06797 -5.44033 0.665177
C -2.99377 -6.3727 0.374023
H -3.83155 -6.54236 1.047347
H -4.56838 -8.4532 -0.30507
C -3.81177 -8.23273 -1.05526
C -3.74036 -8.99082 -2.22175
C -2.76663 -8.69955 -3.18644
C -1.87909 -7.6518 -2.98935
C -1.93357 -6.87635 -1.81441
C -2.91578 -7.18142 -0.82665
H -1.13436 -7.41693 -3.74032
H -2.70455 -9.29435 -4.0938
H -4.43997 -9.80719 -2.38147
H -2.11131 -4.85679 1.579807
H -0.50599 -4.22169 -0.19632
N 0.327826 -6.02494 0.581916
O 0.84237 -5.3926 1.48379
O 0.553433 -7.17359 0.266298

^{4}Int 2$_{B}$

Co -1.48007 -4.74728 -4.23415
N -0.21211 -5.44146 -2.69037
C 0.951195 -4.68565 -2.54813
O 1.796577 -4.8732 -1.6849
C -3.32377 -3.81527 -2.75014
O -2.21011 -3.31541 -2.4463
O -3.42018 -4.64941 -3.72277
C -1.80236 -5.41714 -6.61311
O -1.33632 -6.16901 -5.67812
O -2.08283 -4.2137 -6.37482

C 1.124415 -3.62678 -3.5978
C 2.261602 -2.82307 -3.61375
C 2.35763 -1.82147 -4.57721
C 1.31403 -1.65895 -5.4869
C 0.208798 -2.50488 -5.40987
N 0.122545 -3.46682 -4.48268
H 3.034259 -2.99449 -2.87213
H 3.230816 -1.17588 -4.61497
H 1.347413 -0.88851 -6.25124
H -0.62983 -2.43823 -6.09546
C -2.02678 -6.02205 -7.97844
H -2.17823 -5.24201 -8.72673
H -2.92182 -6.65426 -7.9387
H -1.1829 -6.66179 -8.25409
C -4.56684 -3.48175 -1.95805
H -5.46893 -3.79758 -2.486
H -4.60017 -2.40876 -1.75016
H -4.51418 -4.00509 -0.99711
C -0.88382 -5.90523 -1.65616
C -0.71308 -5.31135 -0.28185
C -1.92859 -5.37216 0.580127
C -2.94553 -6.19806 0.290553
H -3.81204 -6.24944 0.946265
H -4.75464 -8.08178 -0.37605
C -3.94758 -7.99565 -1.10015
C -3.92912 -8.81475 -2.22647
C -2.89041 -8.70611 -3.1578
C -1.88533 -7.76697 -2.97065
C -1.89539 -6.92669 -1.84423
C -2.93495 -7.05398 -0.88276
H -1.08865 -7.67067 -3.69882
H -2.87011 -9.3485 -4.03328
H -4.72442 -9.53959 -2.38017
H -1.92345 -4.75517 1.473049
H -0.30009 -4.30623 -0.33082
N 0.413681 -6.12033 0.51933
O 0.969467 -5.49704 1.401531
O 0.575965 -7.28903 0.229569

[7] References:

[1] S. Rej and N. Chatani, *ACS Catal.*, 2018, **8**, 10949-10949.

[2] D. Guan, L. Wang, H. Song, W. Chu and Z. Sun, *Chem. Lett.*, 2015, **44**, 743-745.

[3] J. Lan, H. Xie, X. Lu, H. Jiang and W. Zeng, *Org. Lett*., 2017, **19**, 4279-4282.

[4] C. J. Whiteoak, O. Planas, A. Company and X. Ribas, *Adv. Synth. Catal.*, 2016, **358**, 1679-1688.

[5] Z.-Y. Jiang, M.-C. Lu, L.-L. Xu, T.-T. Yang, M.-Y. Xi, X.-L. Xu, X.-K. Guo, X.-J. Zhang, Q.-D. You and H.-P. Sun, *J. Med. Chem*., 2014, **57**, 2736-2745.

[6] D. Yasuda, M. Nakajima, A. Yuasa, R. Obata, K. Takahashi, T. Ohe, Y. Ichimura, M. Komatsu, M. Yamamoto, R. Imamura, H. Kojima, T. Okabe, T. Nagano and T. Mashino, *Bioorganic Med. Chem. Lett.*, 2016, **26**, 5956-5959.

[7] F. Neese (2018), Software update: the ORCA program system, version 4.0. WIREs Comput. Mol. Sci., 8: e1327. doi:10.1002/wcms.1327.

[8] F. Neese, *J. Comp. Chem.*, 2003, **24**, 1740.

[9] S. Grimme, S. Ehrlich and L. Goerigk, *J. Comp. Chem*. 2011, **32,** 1456-1465.

[10] (a) F. Weingrad and R. Aldrichs, *Phys. Chem. Chem. Phys.***,** 2005, **7**, 3297. (b) A. Schaefer, H. Horn and R. Aldrichs, *J. Chem. Phys.,* 1992, **97**, 2571.

[11] F. Neese, F. Wennmohs, A. Henson and U. Becker, *Chem. Phys.*, 2009, **356**, 98.

[12] Y. Zhao and D. Truhlar, *J. Chem. Phys*., 2006, **125**, 194101.

[13] S. Sinnecker, A. Rafendran, A. Klamt, M. Diedenhofen and F. Neese *J. Phys. Chem. A.*, 2006, **110**, 2235.

[14] A. R. Allouche, *J. Comp. Chem*., 2011, **32**, 174.

[15] *M.* Hanwell, D.E. Curtis, D.C. Lonie, T. Vandermeersch, E. Zurek and G. R. Hutchison*, J. Cheminform*., 2012, **4**, 17.

[16] (a) C. Riplinger, P. Pinski, U. Becker, E. F. Valeev, and F. Neese, *J. Chem. Phys.*, 2016, **144**, 024109. (b) M. Saitow, U. Becker, C. Riplinger, E. F. Valeev, and F. Neese, *J. Chem. Phys.*, 2017, **146**, 164105.

[17] M. Sparta and F. Neese, *Chem. Soc. Rev.,* 2014, **43**, 5032-5041.

[18] T. Lu and F. Chen, *J. Comput. Chem*., 2012, **33**, 580-592.

[19] A. J. Bridgeman, G. Cavigliasso, L. R. Ireland and J. Rothery, *J. Chem. Soc., Dalton Trans*., 2001, 2095-2108.

[20] T. Lu and F. Chen, *J. Phys. Chem. A*., 2013, **117**, 3100-3108.

[21] I. Mayer and P. Salvador, *Chem. Phys. Lett*., 2004, **383**, 368-375.
